# Porphene and porphite as porphyrin analogs of graphene and graphite

**DOI:** 10.1038/s41467-023-41461-w

**Published:** 2023-10-09

**Authors:** Thomas F. Magnera, Paul I. Dron, Jared P. Bozzone, Milena Jovanovic, Igor Rončević, Edward Tortorici, Wei Bu, Elisa M. Miller, Charles T. Rogers, Josef Michl

**Affiliations:** 1 Department of Chemistry, University of Colorado, Boulder, CO 80309 USA; 2 Institute of Organic Chemistry and Biochemistry, Czech Academy of Sciences, Prague, Czech Republic; 3 Department of Physics, University of Colorado, Boulder, CO 80309 USA; 4 ChemMatCARS, University of Chicago, Lemont, IL 60439 USA; 5 Chemistry and Nanoscience Center, National Renewable Energy Laboratory, Golden, CO 80401 USA; 6 Renewable and Sustainable Energy Institute (RASEI) at the University of Colorado, Boulder, CO 80303 USA

**Keywords:** Surface assembly, Polymer synthesis, Electronic materials

## Abstract

Two-dimensional materials have unusual properties and promise applications in nanoelectronics, spintronics, photonics, (electro)catalysis, separations, and elsewhere. Most are inorganic and their properties are difficult to tune. Here we report the preparation of Zn porphene, a member of the previously only hypothetical organic metalloporphene family. Similar to graphene, these also are fully conjugated two-dimensional polymers, but are composed of fused metalloporphyrin rings. Zn porphene is synthesized on water surface by two-dimensional oxidative polymerization of a Langmuir layer of Zn porphyrin with K_2_IrCl_6_, reminiscent of known one-dimensional polymerization of pyrroles. It is transferable to other substrates and bridges μm-sized pits. Contrary to previous theoretical predictions of metallic conductivity, it is a p-type semiconductor due to a predicted Peierls distortion of its unit cell from square to rectangular, analogous to the appearance of bond-length alternation in antiaromatic molecules. The observed reversible insertion of various metal ions, possibly carrying a fifth or sixth ligand, promises tunability and even patterning of circuits on an atomic canvas without removing any π centers from conjugation.

## Abstract (web summary)

Here the authors report Zn porphene, a member of a new organic metalloporphene family. Similar to graphene, these also are fully conjugated 2D polymers, but are composed of fused metalloporphyrin rings.

## Introduction

**Fig. 1:**
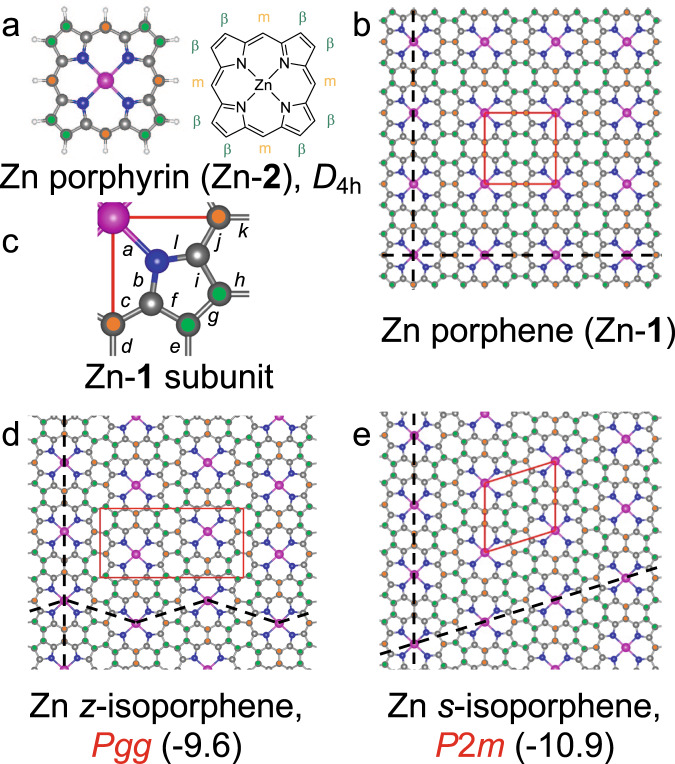
Optimized DFT/PBE50 structures of Zn salts. In red, symmetries: **a** Zn porphyrin (Zn-**2**); **b** Zn porphene (Zn-**1**); **c** bond length map for *P*2*mm* and *P*4*mm* forms of Zn-**1** (cf. Table 1); **d** Zn *z*-isoporphene; **e** Zn *s*-isoporphene. In parentheses, calculated energies relative to Zn-**1** (kcal/mol.macrocycle). Black, green, and red circles are α, β, and meso carbon atoms, respectively; nitrogen atoms are blue, and larger purple circles are zinc atoms. In free bases **1** and **2**, they represent two diagonally disposed H atoms. Red lines define unit cells: Zn-**1**: 8.26 Å × 8.36 Å (*P*2*mm*) or 8.29 Å × 8.29 Å (*P*4*mm*).

Two-dimensional (2D) organic materials^1–13^ offer atomic precision for optoelectronics and energy-efficient nanoelectronics, but most are not easily patterned and tuned. The long-sought^14–22^ porphene [(C_20_N_4_H_2_)_∞_, **1**] is formed in a hole-doped form from the zinc salt C_20_N_4_H_12_Zn (Zn-**2**) of porphyrin (C_20_N_4_H_14_, **2**) by oxidative polymerization on aqueous surface accompanied by loss of Zn^2+^ ions. After hole removal by addition of excess reductant to the subphase, metal ions can be introduced to form Zn porphene, (C_20_N_4_Zn)_∞_ (Zn-**1**, Fig. 1, Table 1), or other metalloporphenes. Reversible insertion of metal ions promises painting on an atomic canvas with distinct metal ions and ligands without removing any π centers from conjugation. The bond pattern in **1** and Zn-**1** is deduced from in situ and ex situ spectra and images. Early density functional theory (DFT) computations for a perfect sheet of Zn-**1** used no Hartree-Fock exchange in the functional and predicted a *P*4*mm* (*D*_4*h*_) square unit cell and metallic conductivity^14,15^, but hybrid DFT predicts a semiconductor with two identical but mutually orthogonally rotated slightly rectangular antiaromatic *P*2*mm* (*D*_2*h*_) unit cells with planar 8-membered rings, analogous to “Kekule” structures of a 2 × 2 fragment of Zn-**1**^23,24^ and planar [4*n*]annulenes^25,26^ (cf. a vast physics literature on 2D-Peierls distortions^27–30^). The polymer sheet was transferred to solid substrates, mostly as multilayers of **1** (porphite) and semiconducting Zn-**1** (Zn-porphite), analogous to graphite, but also as a monolayer.

**Table 1:** Zn-**1**: calculated lengths (in Å) of bonds labeled in Fig. 1

Bond label	*P*2*mm* (0.0 kcal/mol)	*P*4*mm* (3.7 kcal/mol)
*a*	2.02	2.00
*b*	1.33	1.35
*c*	1.40	1.40
*d*	1.41	1.43
*e*	1.36	1.39
*f*	1.45	1.42
*g*	1.42	1.39
*h*	1.40	1.39
*i*	1.38	1.42
*j*	1.41	1.40
*k*	1.28	1.34
*l*	1.39	1.35

Initial approaches to two-dimensional (2-D) organic polymers at liquid interfaces^31^ or surfaces^32–36^ were disappointing; the products were either many atoms thick^31,32^ or small and irregular^33,34^. Only the method they introduced for surface-to-surface transfer by coating with a thin layer of polystyrene, dissolved after transfer^33,34^, is still in use^37^. More recently, sizeable 2-D polymer sheets have been prepared successfully on liquid surfaces^38–43^.

Our preparation of porphene (**1**) by oxidative polymerization of zinc porphyrin (Zn-**2**) in a Langmuir layer was inspired by publications from the groups of Osuka^23,44–46^ and of Vorotyntsev and Devillers^47,48^. The former oxidatively fused derivatized Zn porphyrins into tape- and square-shaped fragments of Zn-**1** with stabilized edges, and the latter anodically oxidized Mg-**2** in bulk solution to a thick layer of charge-transporting polymer of undetermined structure.

Despite the prediction of metallic conductivity and superconductivity for the fully conjugated 2-D sheets of **1** and Zn-**1**^15^, little experimental activity has been directed to their synthesis. We are only aware of one preliminary claim of preparation of μm-sized flakes of **1** ringed with dibromophenyl groups using a different approach, condensation of a dipyrromethane with formaldehyde followed by oxidation^49^.

Oxidative coupling of **2** or its metal salt requires the generation of 12 C–C bonds around the periphery of every porphyrin unit, each with a loss of two protons. At first sight, the coupling^47,48^ is likely to produce an irregular 3-D cross-linked jumble of variously connected porphyrins. Osuka constrained fusion to a limited number of directions through blocking selected edges of starting porphyrins with substituents, but in the synthesis of **1** all four sides of each macrocycle must be free to couple. The mechanism implied by his group’s work^44^ is initial formation of a radical cation, followed by reversible aromatic substitution^50–52^ on an uncharged neighbor, loss of a proton, oxidation of the resulting radical to a cation, and another deprotonation. Similar possibly irreversible events then establish the remaining bonds that finally fuse the edges. Given the reversibility of the initial C–C coupling^53^, we hoped that the anticipated 2-D confined reactions in a pre-organized layer offer only limited choices to each additional molecule of **2**, favoring planarity and repair of defects.

Randomness is a threat even in a 2-D product, because oxidative porphyrin dimerization by edge-to-edge fusion often follows two competing pathways, “m-m + 2×β-β” (three new C–C bonds in a dimer) and “β-β + 2×β-m” (two new C–C bonds in a dimer)^44,54^, sometimes in a random sequence of fusion modes^55^. Formation of **1** requires m-m + 2×β-β fusion at all four edges. Our DFT calculations suggest that regular fusion via m-m + 2×β-β at two opposite edges and β-β + 2×β-m at the other two will be followed by a strongly exothermic Woodward-Hoffmann allowed electrocyclic reaction forming two 7-membered rings and two allylic CH bonds, susceptible to oxidative loss of two more hydrogens to yield many isomers of **1**, such as *s*-isoporphene with straight stripes of Zn⋯Zn centers, *z*-isoporphene with a zig-zag pattern (Fig. 1), and others with more complex patterns and larger unit cells. A β-β + 2×β-m fusion at all four edges would produce a highly strained non-planar isomer. It is unlikely to proceed to the end and such polymer would remain partially hydrogenated, in contrast to what we observe.

Here we describe the preparation of **1** and Zn-**1** by two-dimensional oxidative polymerization of Zn porphyrin on water surface. They are transferable to other substrates. Zn-**1** bridges μm-sized pits and is a p-type semiconductor. A predicted Peierls distortion of its unit cell from square to rectangular is analogous to the appearance of bond-length alternation in antiaromatic molecules. Various metal ions, possibly carrying one or two axial ligands, can be inserted reversibly, promising tunability and patterning on an atomic canvas, yet keeping all π centers in conjugation.

## Results and discussion

**Fig. 2:**
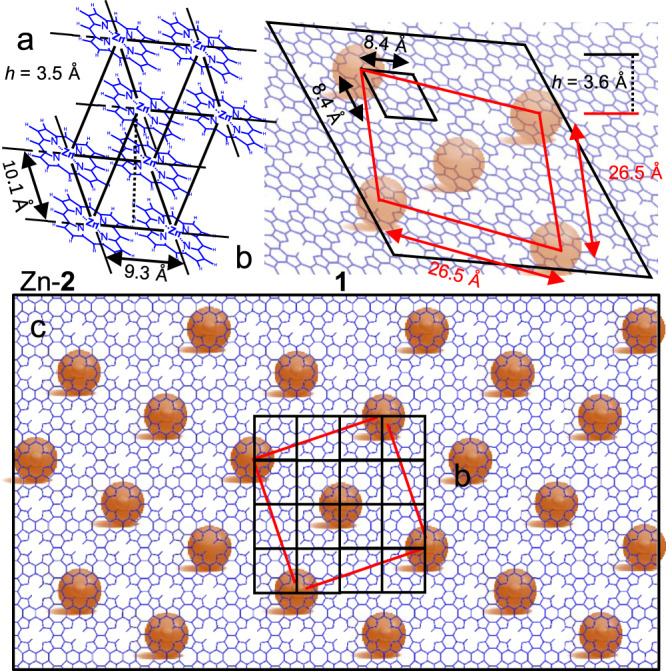
Unit cells at air/water interface from GIXD. **a** Zn-**2**, **b** oblique, and **c** top view of **1** (orange balls: rotationally disordered IrCl_6_^-2^ dianions).

**Fig. 3:**
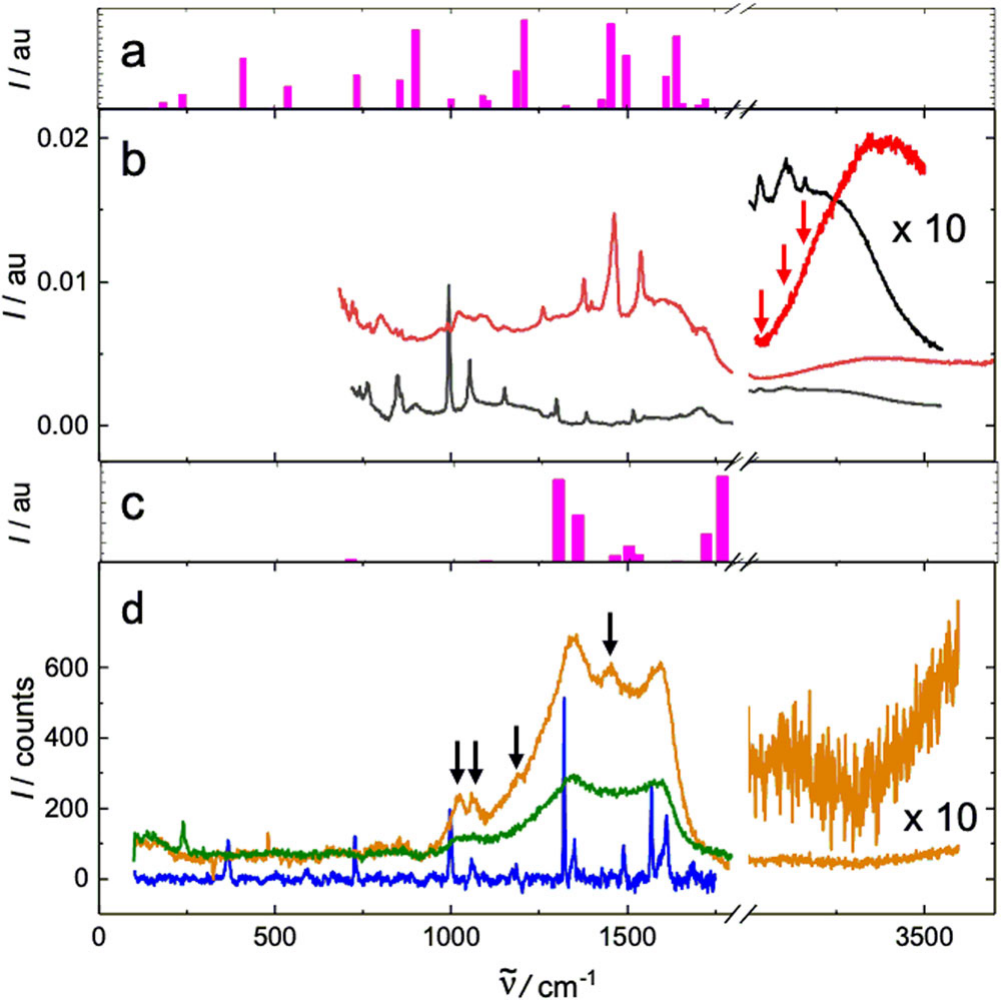
Vibrational spectra of Zn-1. **a** Calculated IR. **b** IR observed on a Ge internal multireflection plate (red, spectrum of Zn-**1**; in black, spectrum of Zn-**2**). **c** Calculated Raman. **d** Raman observed on CaF_2_ (orange, sample polymerized under standard conditions; green, sample polymerized exhaustively; blue, spectrum of Zn-**2**). Black arrows mark defect peaks and red arrows emphasize the absence of CH stretching vibrations associated with defects.

**Fig. 4:**
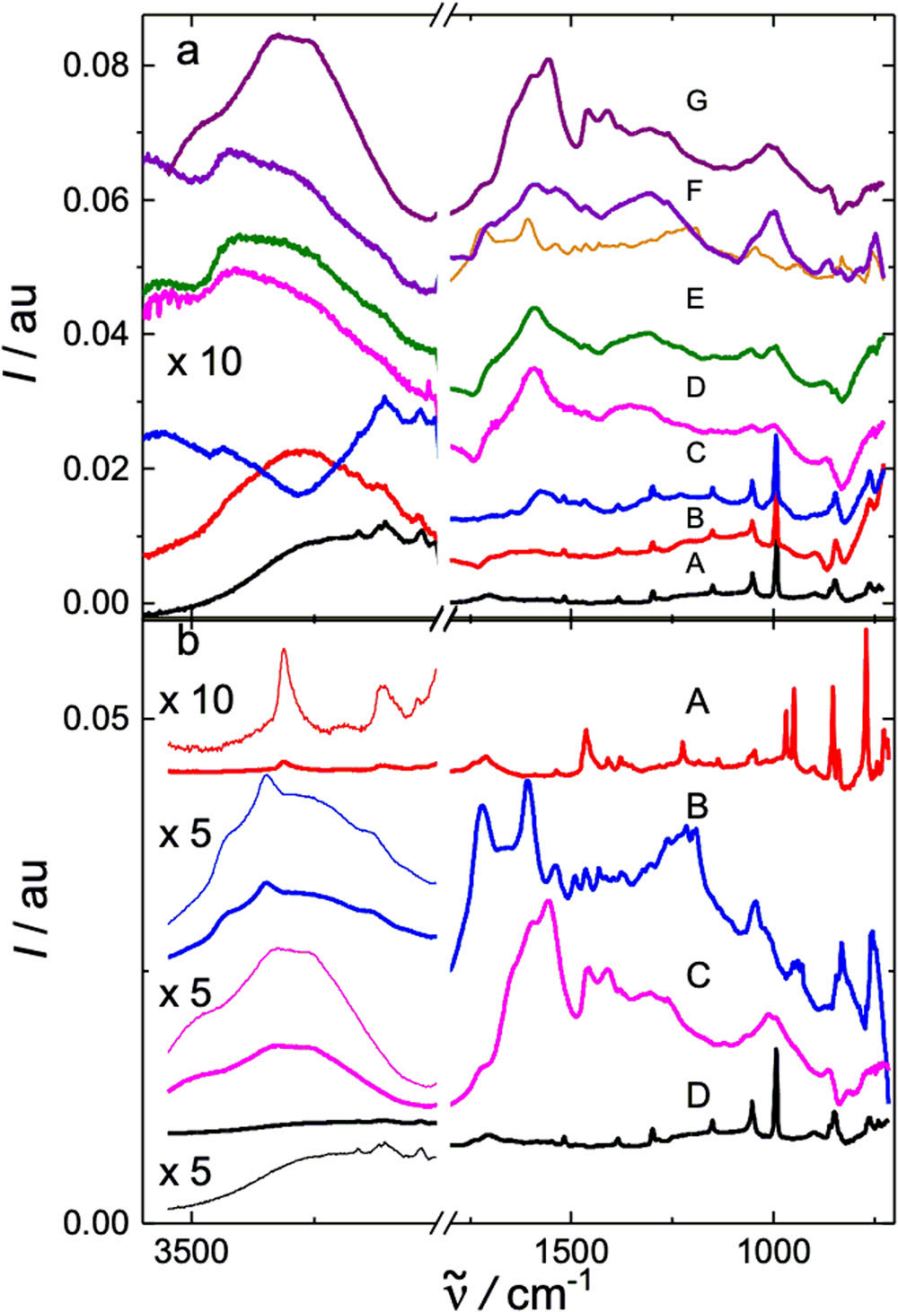
Multi-internal reflection IR spectra. **a** LB layer of Zn-**2** after 3 h of oxidation to **1** with an aqueous subphase containing approx. (A) 0, (B) 0.5, (C) 3.5, (D) 5.3, (E) 7.0, (F) 17.5, and (G) 35.0 μM K_2_IrCl_6_, followed by charge quenching with excess NaI and transfer to Ge. Unlabeled orange curve: contribution of **1** to spectrum (F). The sequence (A)–(F) shows the gradual conversion of Zn-**2** to **1**. Spectrum (G) was taken 24 h after adjusting the subphase to ~0.1 M ZnCl_2_ and pH = 6 and is a spectrum of Zn-**1**. Intensity above 1630 cm^-1^ reflects variable film water content. **b** IR spectrum of a transferred layer: (A) **2**, (B) **1**, (C) Zn-**1**, (D) Zn-**2**.

In a day or less, fresh 0.01–10 mM K_2_IrCl_6_ in the aqueous subphase converts a Langmuir layer of Zn-**2** or Zn-**2**-*d*_12_ into a monolayer of **1** with a loss of all zinc (Fig. 2) and nearly all CH or CD bonds (Figs. 3 and 4), and without detectable formation of isoporphenes. Full removal of CH or CD bonds takes time (see below). Old or more dilute solutions leave some of the zinc inside. In the absence of an oxidant, no loss of zinc is observed from the bilayer of Zn-**2**. We refer to the use of fresh aqueous 0.035 mM K_2_IrCl_6_ for 24 h as “standard conditions”.

**Fig. 5:**
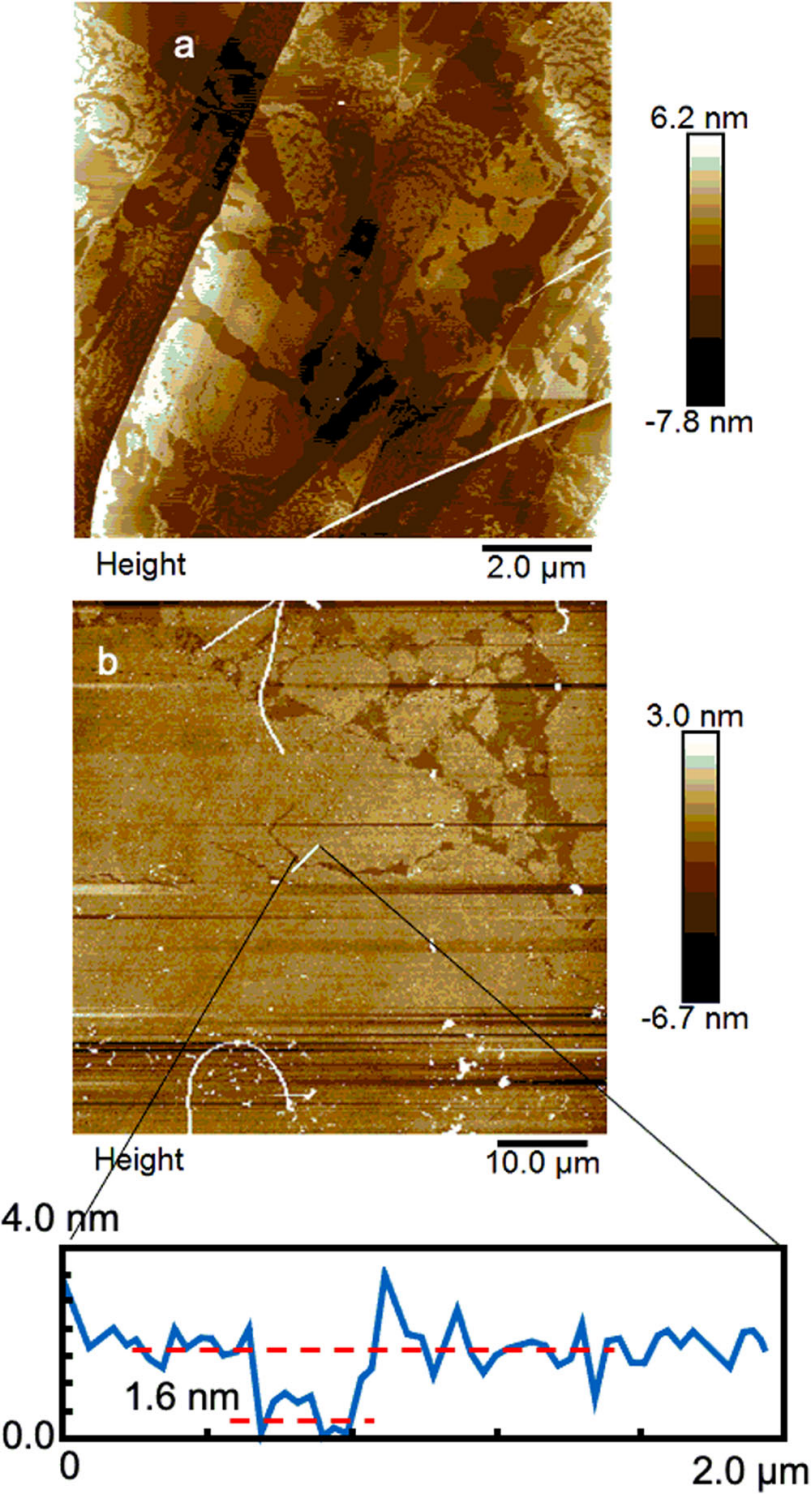
AFM images of 1. Obtained by oxidative polymerization of Zn-**2** under standard conditions and transferred to **a** HOPG and **b** Ge. Bottom: height profile through the white line in (**b**).

**Fig. 6:**
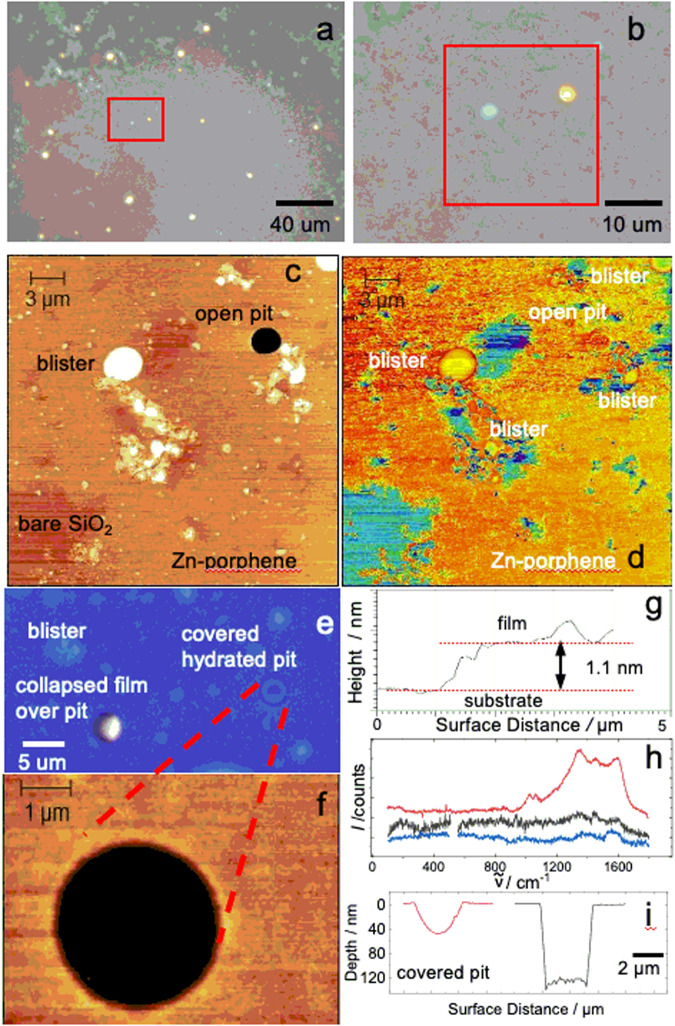
Mechanical strength of monolayer Zn-1 transferred to pitted 120 nm layer of SiO_2_ on Si. **a** Optical microscope image. **b** Enlarged area shown in red box in (**a**). **c** Tapping-mode AFM image of area in red box in (**b**). **d** Phase image of (**c**). **e** Optical microscope image of collapsed film over pit (white and brown color) and covered hydrated pit (blue with dark ring). **f** AFM image of blue-colored covered pit in (**e**). **g** Height profile across the step transition between SiO_2_ bare and covered with film found in the lower left corner of (**c**) and (**d**). **h** Raman spectrum of Zn-**1** on multilayer coated BaF_2_ (red), over a covered blister (black), and over a hydrated pit (blue) shown in (**c**–**e**). **i** AFM profiles for covered hydrated pit (red) shown in (**e**, **f**) and uncovered pit (black).

**Fig. 7:**
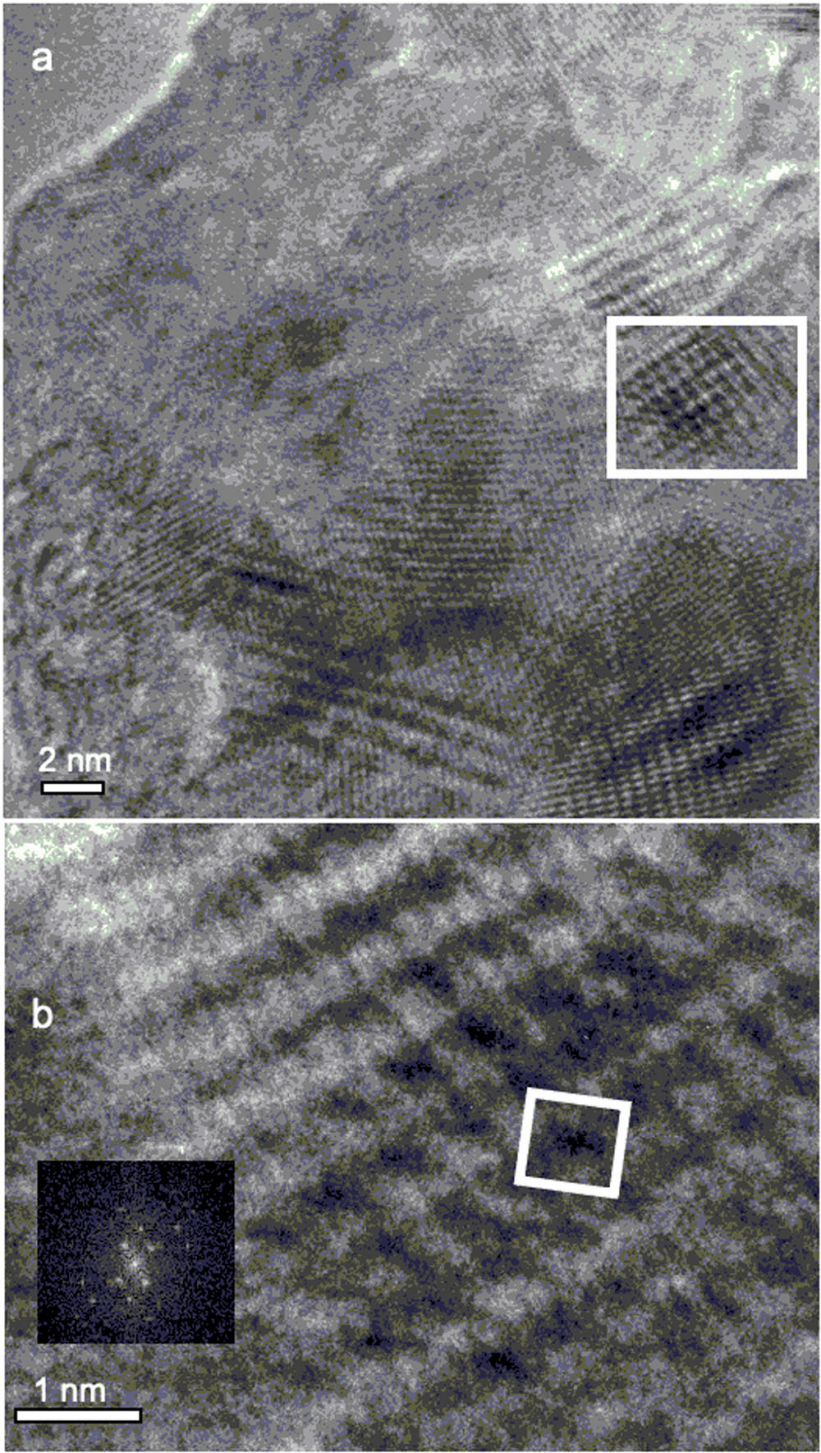
TEM images. **a** Zn-porphite polymerized under standard conditions and transferred to Au mesh. **b** Enlargement of the white box shown in panel (**a**), with a 8.4 × 8.4 Å^2^ unit cell deduced for **1** from GIXD and a (0.5,0.5) offset between layers. Inset shows FFT of (**b**).

**Fig. 8:**
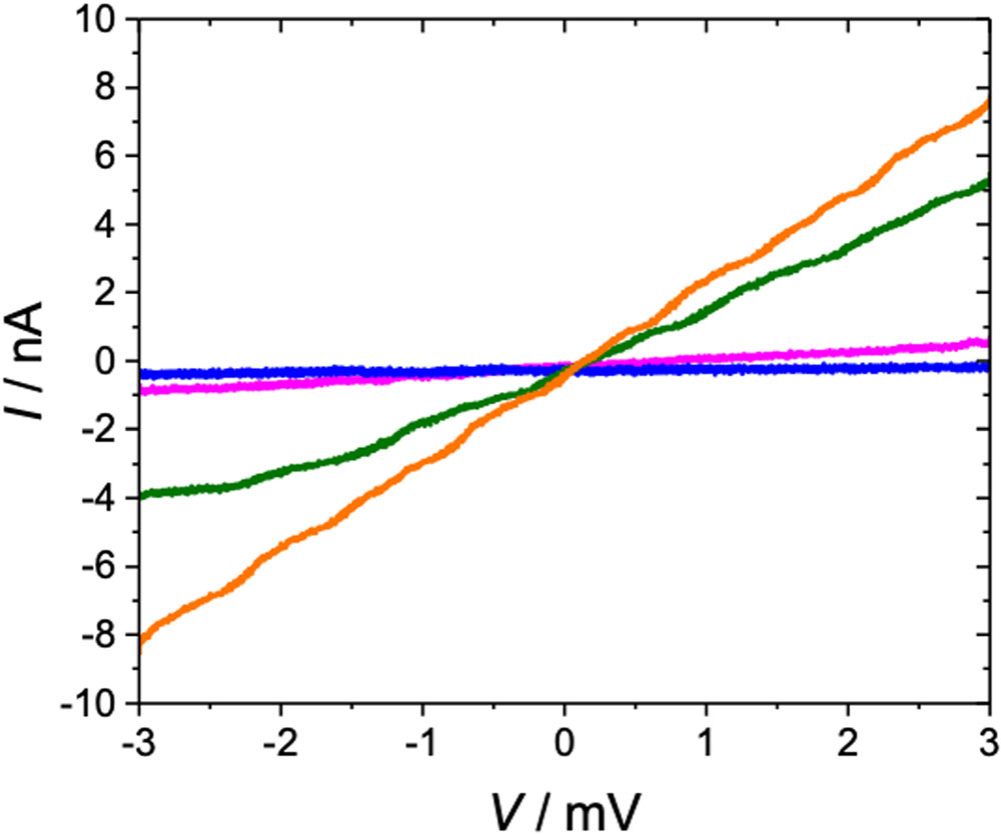
*I–V* curves for multilayer Zn-1. Prepared under standard conditions and doped under a saturated I_2_ hexane solution after 0 min (blue), 1 min (magenta), 3 min (green), and 5 min or longer (orange).

Like graphene, **1** is gray (Supplementary Fig. 1), sturdy (Figs. 5 and 6), transferable (cf. the ex situ section below), self-supporting (Figs. 6 and 7, Supplementary Figs. 2 and 3), and transfer to solid substrates can be directed to build monolayers (Fig. 6 and Supplementary Fig. 2) or multilayers analogous to graphite (Fig. 7). We have not detected the metallic conductivity predicted^15^ for a perfect infinite sheet but conductivity was induced reversibly by doping with I_2_ in hexanes (Fig. 8).

Structural evidence for oxidative polymerization of Zn-**2** to form **1** was obtained both in situ (on water, before or after Zn^2+^ reintroduction) and ex situ (after Zn^2+^ reintroduction and transfer to a solid substrate). (i) In situ measurements used UV-visible spectroscopy (UV-vis, Supplementary Fig. 4A), grazing incidence X-ray diffraction (GIXD, Fig. 2 and Supplementary Fig. 5D–F), and X-ray reflectivity (XR, Supplementary Fig. 5H), and after conversion to Zn-**1**, also Brewster angle microscopy (BAM) and Langmuir isotherms (Supplementary Fig. 5B, C). (ii) Ex situ measurements used ultraviolet-visible-infrared (UV-vis-IR, Supplementary Fig. 1A), mid-infrared (IR, Figs. 3 and 4), resonance Raman (Fig. 3), and X-ray photoelectron spectroscopy (XPS, Supplementary Fig. 6), and atomic force (AFM, Fig. 5) and transmission electron (TEM, Fig. 7) microscopy. Details of the structure proof are provided in Supplementary Notes.

Like all previous cyclic boundary conditions periodic DFT calculations for porphenes, ours suffer from the use of a single reference configuration at or near *P*4*mm* geometries where at least two are called for. In principle, a single reference configuration would be adequate if the quality of the treatment were close to full configuration interaction, but with 25 atoms in the unit cell, this is presently beyond reach. A hybrid functional with ≥25% exact exchange and a geometry optimized to rectangular *P*2 (Fig. 1) nevertheless provides overall compatibility with experimental observations, including the absence of metallic conductivity (Fig. 8).

Results of the in situ measurements are considered first.

### UV-vis spectra

Supplementary Fig. 4A provides evidence for a slow chemical transformation in the LB layer of Zn-**2** by K_2_IrCl_6_ in the subphase. The Soret band is first replaced by broad absorption at lower energies expected^45^ for edge-fused porphyrins, some of which disappears again at very long times (see ex situ studies below).

### LB isotherms and Brewster angle microscopy

See Supplementary Fig. 4B, C and Supplementary Note 1. Porphyrins usually yield poorly defined Langmuir isotherms^56^ (surface pressure as a function of mean area per molecule, mmA). On pure water, the isotherm of Zn-**2** rises perceptibly above baseline at mmA of 80−90 Å^2^/macrocycle and steeply at mmA of ~50 Å^2^/macrocycle. Calculated molecular footprints are ~108 Å^2^/macrocycle with molecules flat on the surface and ~35 Å^2^/macrocycle when nearly perpendicular to it (Supplementary Fig. 7). Most likely, at high dilution Zn-**2** molecules lie flat on the surface and upon compression slide over each other until they produce a full bilayer, which resists further compression. BAM reveals small highly scattering islands, presumably Zn-**2** microcrystals, moving slowly independently of each other, probably due to air currents. After polymerization to **1**, they are still present and are interspersed with large zones of reduced contrast, but their motions are correlated, all as expected for a surface covered with large rigid sheets (Supplementary Fig. 4C).

After polymerization, the onset of a steep rise upon compression is poorly reproducible. For both **1** and Zn-**1** it occurs at mmA 65–105 Å^2^, presumably depending on how the large sheets happen to pack. The mmA/macrocycle value expected at perfect monolayer packing both from GIXD and DFT is 71 Å^2^. It is thus highly unlikely that **1** could be present as a bilayer, for which a steep rise should occur at mmA of ~35 Å^2^, a value much smaller than any observed in numerous experiments.

### GIXD and XR

See Fig. 2, Supplementary Fig. 5, Supplementary Tables 1 and 2, and Supplementary Note 2. Analysis by previously described methods^57^ showed that the dominant phase occurs in single crystalline domains. Zn-**2** forms pre-organized crystalline bilayer domains with a 3.5 Å interlayer spacing, an almost orthogonal (*θ* = 84.4°) primitive 2-D unit cell (9.3 × 10.1 Å, *P1*, average domain size 38 nm), and slightly twisted (14.4°) fully metallated Zn-**2** macrocycles lying nearly flat (tilt angle 24.8°). The upper layer is offset by 0.5 and 0.08 of the unit cell length along the short and long directions, respectively. The mmA of 46.5 Å^2^/molecule at perfect packing calculated from the unit cell is slightly smaller than the ~50 Å^2^/molecule found from the Langmuir isotherm, a difference attributable to a minor amount of Zn-**2** microcrystals observed by BAM, or of other unknown material.

For **1** an examination of many possible structures identified only one unit cell that fits the observed GIXD pattern (Supplementary Fig. 5F). This is the porphene cell shown in Fig. 2. It is compatible with all other evidence, and positions macrocycle centers 8–9 Å apart as required by the one or more CC bonds connecting their edges. Others were dismissed because they disagreed with the XR results or required a bilayer of **1**, which would contradict the LB isotherm. Numerous attempts to fit the GIXD pattern to *z-* or *s*-isoporphene structures (Fig. 1de) failed. The considerable difference between the diffraction patterns expected for **1** and for these isomers is exemplified in Supplementary Fig. 8.

The acceptable solution we found is a chiral superlattice of a square or approximately square grid monolayer of **1** with a unit cell of 8.4 ± 0.1 Å length (90°, *P*4*mm*, average domain size >123 nm), containing a single macrocycle, located 3.6 Å above a second square lattice with a 26.5 Å unit cell (90°, *P*4*mm*, average domain size 19 nm), containing two rotationally disordered IrCl_6_^−2^ ions. The lattice vectors of the two cells are not aligned and the Bravais lattice is monoclinic. The best fit is found if the anion lattice is non-primitive and 2-D body centered with one corner offset relative to the polymer unit cell by (0.5,0) and twisted by 18.5° (Fig. 2). The footprint is 73.0 Å^2^ mmA/macrocycle. This solution agrees with the observed XR (Supplementary Fig. 5H) and with the LB isotherm (Supplementary Fig. 4B). We cannot tell how the -NH- and =N- groups are arranged in the free-base macrocycles nor how many macrocycles are oxidized to radical cations. If the **1**/IrCl_6_^−2^ superlattice is electroneutral, the oxidized fraction is 40%. If the superlattice carries a net charge, it will be compensated by disordered ions in the nearby layer of solution. Precedents for such situations exist^58,59^.

The resulting structure was compared with results of DFT geometry optimizations for Zn-**1** (Fig. 1), which depend on the functional used. Calculations without any Hartree-Fock exchange, known to exaggerate delocalization^60^, agree with earlier reports^14,15^ and yield a single *P*4*mm* minimum (8.42 Å × 8.42 Å for PBE) with partially filled bands, suggesting metallic conductivity. For hybrid functionals with ≥25% of Hartree-Fock exchange, there are two potential energy minima at mutually perpendicular slightly rectangular *P*2*mm* geometries (8.26 Å × 8.36 Å for PBE50), and the band structure is that of an indirect band semiconductor. Both results agree with the structure deduced from the in situ GIXD pattern within its uncertainty limits (the PBE calculated 4.0 Å interlayer separation is excessive). It is presently not known whether the heavy hole doping and the presence of water and counterions affect the observed structure.

At the *P*2*mm* geometry, optimized with functionals containing ≥25% of exact exchange, the energy of **1** is calculated to be a few kcal/mol.macrocycle below that of the *P*4*mm* geometry. The difference increases as the amount of exact exchange grows. The results for **1** and Zn-**1** are similar and those obtained with an atomic and a plane wave basis set agree (Supplementary Table 3).

Next, we address the results of ex situ measurements (Supplementary Note 3).

### Vibrational spectra

Oxidative polymerization of Zn-**2** was monitored either by performing the NaI quench at various times after the reaction was launched or by keeping the reaction time constant and varying the concentration of K_2_IrCl_6_. Similar experiments were performed with Zn-**2**-*d*_12_. Concentrations up to 0.21 M and reaction times up to 120 h were used to obtain complete conversion (Fig. 3). To optimize the signal to noise ratio, the Zn-**1** monolayer was transferred to substrates at a surface pressure high enough to convert it into small domains of bilayers and multilayers (Zn-porphite). The IR spectra (Fig. 3) simultaneously allow monitoring the loss and reinsertion of Zn^2+^ in **1**, using its NH stretch at 3340 cm^−1^ (cf. 3309 cm^−1^ in **2**) and NH pyrrole deformation at 1227 cm^−1^ (cf. 1223 cm^−1^ in **2**).

Figure 4 shows the IR and Supplementary Fig. 9 the resonant Raman spectra of incompletely oxidized samples. Supplementary Fig. 10 compares the Raman spectra obtained starting with Zn-**2**-*h*_12_ and with Zn-**2**-*d*_12_. Supplementary Fig. 11 contains the DFT optimized structure of the proposed most common defect due to incomplete oxidation. As deduced from peak positions and integrated intensities in the vibrational spectra, the defect is a porphyrin macrocycle that still has 10 of its original 12 CH or CD bonds, is only attached to two of its neighbors at two opposed meso positions, and is twisted nearly perpendicular to the porphene plane. The overall observed and DFT calculated UV-vis-NIR-IR absorption can be seen in Supplementary Fig. 1, where difference spectroscopy isolates the visible spectrum of the defects. Results of DFT calculations on a simplified nine-porphyrin model for the defect account for this spectrum well and computed transition densities collected in Supplementary Fig. 12 clearly distinguish excitations localized on the twisted macrocycle from those localized in its immediate vicinity where edge fusion failed to occur and which have also kept a total of ten CH or CD bonds.

The key peaks used for the observation of residual CH or CD bonds, identified by their large isotopic shifts, were the stretches at 3034 cm^−1^ (C_m_H) and 3110 cm^−1^(C_β_H) in the IR^61,62^ and the in-plane bends at 1158 cm^−1^ (C_m_H) and 1060 cm^−1^(C_β_H) in the Raman^61,62^ spectrum, assigned in analogy to the vibrations of parent porphyrin (Supplementary Table 4). The standard polymerization conditions (fresh 0.035 mM K_2_IrCl_6_ for 24 h) were chosen as those under which the noise in our IR spectra totally obscured any residual peaks of CH or CD bond vibrations, although the Raman spectra still showed them.

Given the level of noise in the IR spectrum, the ratio of fully incorporated to defective (3.5 × 10^12^/cm^2^) porphyrin macrocycles is at least 40 under the standard synthetic conditions. Under the most forcing conditions, when not even the resonant Raman spectra show any CH or CD bonds above the noise level, the ratio is at least 400 and the defect density is at most 3.5 × 10^11^/cm^2^. These results for identified defects compare well with defect densities observed in other 2-D materials (10^15^/cm^2^ in fully disordered graphene^63^ and 10^13^/cm^2^ in MoS_2_^64^. The presence of other types of defects that are spectroscopically silent cannot be excluded and is likely at domain boundaries. Vibrational spectra thus demonstrate the absence of significant amounts of CH bonds in the polymer and limit its structure to Zn-**2** and zinc isoporphenes (Fig. 1). As noted above, the GIXD results rule out the presence of significant amounts of the latter. The faster disappearance of meso CH bonds than β CH bonds during the synthesis also speaks in favor of the porphene structure.

### Electrical conductivity

See Supplementary Note 4. This probably is the most immediately interesting of all material properties of a previously unknown family of 2D materials. At the moment, we have only examined room-temperature conductivity of multilayer Zn-**1** and found that the undoped material is an insulator and after doping with I_2_ follows Ohm’s law (Fig. 8). It loses conductivity when the dopant is removed.

### XPS

See Supplementary Fig. 6. These results provide independent evidence for the transformation of **1** into Zn-**1**. The Zn(2*p*) peak of Zn-**1** is absent after polymerization, which forms **1**, and is presumably present after Zn^2+^ is reinserted to produce Zn-**1**, but was obscured by ZnCl_2_ impurity. We therefore focus on the N(1*s*) binding energies^65^, which show a strong increase relative to monomeric porphyrins (Table 2, cf. previous reports for **2**^66^ and for zinc salts of substituted porphyrins^67^). A 2.5 eV binding energy difference between Zn-**1** and Zn-**2** is well reproduced by our DFT calculation, which yields a difference of 2.2 eV for the rectangular *P*2*mm* form. A calculation for the square *P*4*mm* form gives a very large value of 4.7 eV, which is not surprising considering that this form is calculated to be metallic, providing very effective hole screening. Next to the observation of low electrical conductivity of undoped Zn-**1**, the much better agreement between the observed and calculated N(1*s*) binding energy difference for the rectangular than the square form provides further evidence for a rectangular unit cell and a semiconductor, and shows that the use of at least 25% of exact exchange in the DFT functional is justified.

**Table 2:** Binding energies (eV) of nitrogen core levels^a^

Compound	N(1*s*)^A^	N(1*s*)^B^
**2**^b^ Zn−**2** obsd.^b^ calcd.^c^	398.2 (0.68, 440) 398.5 (0.80, 231) [400.5 (0.53, 83)] (398.5)	400.2 (0.89, 946) 398.5 (0.80, 231) [400.5 (0.53, 83)] (398.5)
**1**^d^ Zn-**1** obsd.^d^ calcd.^c^ square^e^ calcd.^c^ rect.^f^	399.4 (0.83, 130) 401.0 (0.81, 213) 403.2 400.7	400.7 (0.83, 190) 401.0 (0.81, 213) 403.2 400.7

^a^In parentheses, peak width in eV and relative area. A: Imine nitrogen; B: Pyrrole nitrogen. Data in brackets are attributed to a shake-up satellite.

^b^Cast polycrystalline film on ITO.

^c^Only relative calculated values are meaningful. The computed values have been reduced by 14.7 eV to fit the value observed for Zn-**2**.

^d^LB film on ITO.

^e^Geometry optimization constrained to *D*_4h_ symmetry.

^f^Unconstrained geometry optimization.

### AFM

See Fig. 5, Supplementary Note 5. Transfer to a solid substrate permits AFM imaging of porphite. It illustrates the mechanical strength of the 2-D sheets draped over steps in HOPG, and demonstrates their large size, up to many scores of μm. The conformity of the edges of some of the neighboring patches shows that they were torn by shearing forces in transfer and remained intact thereafter.

### TEM

Zn-**1** converted to Zn-porphite by transfer at high surface pressure shows overlapping lattices and moiré patterns in a disordered collection of small crystalline domains of Zn-**1** multilayers (Fig. 7). The white box in Fig. 7 matches the unit cell expected for Zn-**1** with a (0.5,0.5) offset between neighboring layers. An analysis of Zn⋯Zn distances at various angles of electron incidence agrees with the dimensions determined in situ by GIXD and provides an independent confirmation of the prevalence of m-m + 2×β-β coupling in the formation of **1** from Zn-**2**. The thinnest free-standing layers were rapidly degraded in a 200 keV e-beam of an HRSTEM instrument.

Most of our ex situ observations were made on multilayers of Zn-**1**, which provide better signal-to-noise ratios. However, at lower surface pressures it is possible to transfer patches of monolayers to solid substrates and we have used them for a qualitative test of the mechanical strength of a Zn-**1** monolayer. Figure 6 and Supplementary Fig. 2 show the results obtained by AFM and optical microscopy, respectively, on a Zn-**1** monolayer that has been transferred to an Si substrate covered with a 120 nm layer of SiO_2_ containing micron-sized circular pits in which the SiO_2_ has been removed but which might be filled with water. The polymer is suspended over some of the pits, behaves as a drumhead, and is strong enough that we were unable to break it with the AFM cantilever tip.

### Aromaticity and antiaromaticity

Antiaromaticity of a substituted 2×2 fragment of porphene has been discussed^23^ and it now appears that it can be extended to an infinite polymer sheet. In spite of the weakness related to the use of a single reference configuration, the present periodic boundary condition calculations credibly suggest the existence of a striking difference between the antiaromatic porphenes with two potential energy surface minima in a primitive unit cell and the aromatic graphene with one. If confirmed by higher level calculations and less ambiguous experimental tools, this suggests a general classification of fully conjugated two-dimensionally infinite π-electron systems. Like aromatic annulenes and many more complicated polycyclic molecules, aromatic 2D polymers would be built from units possessing a single equilibrium structure described by a superposition of two strongly interacting Kekule resonance structures. Antiaromatic 2D polymers would be built from two (and possibly more) distinct units, each corresponding primarily to one Kekule structure, interacting only weakly with the other. In Zn-**1**, the source of antiaromaticity is the fully conjugated 8-membered ring, but it is possible that the presence of a heptalene structure in the presently unknown Zn *s*-isoporphenes (Fig. 1e) might make them antiaromatic, too. The lower energy of the *z*- and *s*-isomers may be primarily due to reduced strain in their σ skeleton. It is noteworthy that the polymerization of Zn-**2**, whose first steps are believed to be reversible, nevertheless in the end yields the less stable isomer. Presumably, a reversible meso-meso coupling occurs first and favors the formation of an essentially defect-free hydrogenated m-m coupled precursor to porphene, and subsequent beta-beta coupling is no longer reversible.

The square to rectangle distortion of the unit cell observed in our calculations on porphene is a form of two-dimensional Peierls distortion, much studied by physicists on graphene, e.g., refs. ^27–30^. The very small calculated barrier separating the two nearly square rectangles in Zn-**1** suggests that their interconversion by thermal activation or by tunneling is rapid and that averaged over a long time period, unperturbed Zn-**1** has fourfold symmetry.

### Opportunities for patterning

Ordinarily, metal ions enter porphyrin macrocycles readily and a low pH is required to remove them. The loss of Zn^2+^ ions into the subphase during oxidative polymerization is however not induced by acid^68^, but by the oxidant (bulk pH is ~6 and is not perturbed significantly by the small number of protons produced by the slow surface oxidative polymerization). Since it initiates polymerization by converting Zn-**2** into a radical cation, the oxidant surely also injects positive charges into the more extensively conjugated Zn-**1**, diminishing the binding constant of Zn^2+^ ions. After charge removal with excess NaI, addition of metal cations converts **1** to Zn-**1** and other metalloporphenes readily (ZnCl_2_ is used at pH ~3 to avoid hydrolysis). The method of metallization of **1** appears to be general, judging by IR and XPS results obtained when FeCl_2_ or CuCl_2_ are used instead of ZnCl_2_.

The reversible insertion of metal ions into the macrocycles in **1** offers 2-D patterning without jeopardizing mechanical strength. About 60 different elements have been inserted into monomeric porphyrins^69^ (almost all metals and some non-metals), and can attach two, one, or zero additional ligands. These could be bidentate, stitching two or more sheets into multilayers analogous to metal-organic frameworks. A layer-by-layer fabrication would allow aperiodic variation of dopants and separation of neighboring layers by different functionalized ligands, hence patterning in the third dimension and access to programmable solids^70,71^.

## Methods

### Materials

Porphyrin (**2**) and Zn porphyrin (Zn-**2**) were synthesized^72,73^ or purchased from Frontier Specialty Chemicals, Logan, UT. Porphyrin-*d*_12_ (**2**-*d*_12_) and Zn porphyrin-*d*_12_ (Zn-**2**-*d*_12_) were prepared by a published procedure^74^. K_2_IrCl_6_, NaI, and ZnCl_2_ were obtained from Sigma Aldrich. Water (10 MΩ) was from Millipore.

### Langmuir-Blodgett (LB) trough and Brewster angle microscopy

Two LB troughs with free access to laboratory atmosphere were used at Boulder. One was home built from teflon and the other was a Kibron PTFE minitrough XS equipped with a KSV micro BAM (Biolin Scientific) Brewster angle microscope (field of view, 4.0 × 3.0 mm). Fresh solutions were prepared by sonicating Zn-**2** (0.2 - 0.4 mg) with benzene (25 mL) for at least 30 min. During the polymerization reaction, the mean molecular area (mmA) was kept at 125 - 135 Å^2^/macrocycle except for some experiments at a synchrotron, where it was oscillated as described below. During transfer of the product to a solid substrate, two sets of conditions were used. For maximum product areal density (multilayer transfer) the mmA was 40 - 80 Å^2^/macrocycle. For transfer of a single monolayer by a Schaefer or a 30 deg LB transfer it was 110 - 130 Å^2^/macrocycle.

### Grazing incidence X-ray diffraction and X-ray reflectivity

These measurements were performed using GIXD and XR equipment, experimental methods, and software available at the ChemMatCARS facility at the Advanced Photon Source in Argonne National Laboratory. The procedures have been previously described^57,75^. All measurements were done at room temperature under a He atmosphere that maintained the O_2_ level below 1%.

For measurements with unpolymerized film the subphase was water. A known amount Zn-**2** solution in benzene was applied to the aqueous surface. After evaporation of benzene and establishment of the He purge (~30 min) the film was compressed to a preset mN/m value at a rate of 1 mm/min. For the preparation of polymerized films the K_2_IrCl_6_ oxidant was present in the subphase. After the He purge was established the barriers were cycled for 1−12 h between two selected barrier positions determined by a pair of chosen mean molecular areas (mmA). Prior to the GIXD measurements, the cycling was stopped and the barriers were moved to obtain either a desired mmA or a desired surface pressure, which was then maintained during the measurement. The same results were obtained when the oxidative polymerization was performed at a constant mmA of 125 - 135 Å^2^/macrocycle.

The beam energy was 10 keV (λ = 1.23984 Å) with a resolution-limited diffraction peak with full width at half maximum (FWHM) *ΔQ*^*exp*^_*xy*_ of 0.0134 Å^−1^ for the slit combination along the detector beam. Domain size was obtained from the diffraction peak FWHM corrected using *ΔQ*^*Corr*^_*xy*_ = (*ΔQ*^*exp*^_*xy*_^2^ + *ΔQ*^*inst*^_*xy*_^2^)^½^, where *ΔQ*^*exp*^_*xy*_ and *ΔQ*^*inst*^_*xy*_ are the observed and instrumental FWHM, respectively.

“Burn” tests, performed to assess the stability of the films to degradation under a continuously purged He atmosphere, indicated no degradation of Zn-**2** after 6 min in the beam. A step-and-collect procedure was used to minimize the cumulative damage in each beam spot with the trough translated under the beam by a few mm to insure a fresh spot at the start of each diffraction or reflectivity scan.

### UV-visible absorption spectroscopy

In situ UV-visible spectra of LB films were measured in the sample compartment of a Cary 2000 UV-vis-NIR spectrometer fitted with a home-built Langmuir trough. The sample beam of the spectrometer was deflected through the water surface and back with a series of mirrors, one of which was submerged in the trough. The sample beam probed the film side of the trough barrier. An identical arrangement was used for the reference beam which was deflected through the water surface on the film-free side of the barrier. Difference spectra were recorded by using as a reference background the spectrum of the porphyrin before polymerization. After this was recorded, the oxidant was introduced to the subphase. The visible absorption due to IrCl_6_^−2^ and IrCl_6_^−3^ changes in time, presumably because the former oxidizes water. The reference beam monitored these extinction changes and automatically removed them from the successively taken spectra of the polymerizing film. The Cary 2000 permits monitoring the single beam intensity and the water level in the trough was adjusted periodically over the course of hours to return the reference beam intensity to its initial level by replacing water lost to evaporation. The parameters for making the film and polymerizing it were similar to those described above and chosen for a system with an mmA of 100 Å^2^/macrocycle.

Ex situ spectra of LB films of Zn-**1** from 250 nm to 5 μm were obtained on a CaF_2_ or BaF_2_ window. An InSb detector was used for the segment extending into the mid-IR. This segment was normalized to match the NIR end obtained in the Cary 2000 or 3000. The sample was baked to remove water trapped between the film and substrate, which produces a broad peak near 3000 nm.

### Infrared absorption spectroscopy

IR spectra were measured with a Nicolet Nexus 670 spectrometer after transfer of films to a Ge multi-internal-reflection (MIR) plate (20 reflections) at 4 mm/min after compression to a surface pressure above 5 mN/m at a speed of 4 mm/min. The transfer ratio is variable; the average value was 75%. The relatively high pressure is optimized for the transfer ratio and not to maintain the integrity and orientation of the film. The transfer is likely to produce some multilayer stacking of the native film.

The transferred films contain water. A weak stretching band at 3440 cm^−1^ and a strong bending band at 1630 cm^−1^ have been reported for hydrated Ge surface previously^76,77^. The intensity of the observed broad band at ~3500 cm^−1^–~3000 cm^−1^ and peaks at 1630 cm^−1^ varied and was reduced by baking or retention in the dry atmosphere of the FTIR sample compartment. These absorptions only appear in presence of a film and are assigned to complexed and hydrogen-bonded water trapped underneath. When the intensity of the 1630 cm^−1^ band is reduced by drying or baking, the underlying band of **1** appears with a clear dispersive lineshape.

The Ge substrate surface contains somewhat water soluble oxides and hydroxides in variable amounts that make the baseline highly sensitive to minute details of the experimental procedure when combined with a twenty-reflection MIR, even when the treatment is very carefully controlled.

In some instances it was possible to transfer, under high surface pressures (>25 mN/m), multilayer films spanning a few mm to BaF_2_ and CaF_2_ substrates. When such films were available transmission spectra were taken using a thin slit mask whose width matched the multilayer film.

### Raman spectroscopy

A Horiba Labram HR Evolution confocal Raman spectrometer at 532 nm was used to obtain resonance Raman spectra and films transferred to BaF_2_, CaF_2_, or SiO_2_/Si substrates. The films were transferred from the LB trough using multiple dip cycles under high pressure. This made a narrow optically thick band spanning a few to less than one mm across the face of the substrate. To prevent spectral contamination from degradation products low laser power was used (2.5%) with a shoot-and-move data collection procedure. Full laser power applied for tens of seconds led to detectable damage to the film and was avoided.

### X-ray photoelectron spectroscopy

XPS data were obtained with a Kratos AXIS Nova spectrometer, calibrated with Au, Ag, Cu, and Mo metal surfaces, cleaned by Ar-ion sputtering, using Al K_α_ radiation (1486.7 eV), a pass energy of 10 or 40 eV, and a step size of 0.1 eV. Measurements were done on films cast from solution or transferred by the Langmuir-Blodgett (LB) method to indium tin oxide (ITO) substrates. The average ITO composition (atomic weight per cent) was 74.4% In, 8.8% Sn, and 16.9% O and compared well with the nominal batch value of 74%, 8%, and 18% typical of commercial ITO, with the substrate accounting for the entire O(1s) intensity. Cast films were made by evaporation of 10^−4 ^M solutions onto freshly cleaned ITO. LB films were made after compression to 1–2 mN/m. The ITO substrate was pulled upward through the interface at 1 mm/min.

Cubic continuation, Shirley, and Tourgaard^78^ methods of baseline subtraction were tested with little noticeable difference in the fits. The more elaborate Shirley and Tourgaard methods, which correctly account for the population of inelastic electrons ejected from higher energy orbitals into those that are lower, were found to be unnecessary due to atomic thinness of the sample and the relatively few inelastic electrons ejected. They were not used.

### Atomic force microscopy of Zn-1 multilayers

AFM images were obtained with either a Digital Instruments Nanoscope IIIa or Dimension systems. Films were transferred at 75–110 Å^2^ mmA/macrocycle at 3 mm/min, either vertically to Si or Ge substrates or at a transfer angle of ~30°, relative to interface surface to HOPG substrates. For the latter, vertical transfer is inefficient.

### Atomic force microscopy of Zn-1 monolayers (possibly, bilayers)

SiO_2_ (120 nm) was thermally grown on an Si wafer, coated with a negative resist (AZ nLOF 2035), and “hard baked” at 160 °C for 10 min. Imperfections in the resultant resist coating were CF_4_ plasma etched into randomly sized (1–12 μm) and randomly located 120 nm deep pits. A Langmuir-Blodgett film of Zn-**1** prepared under standard conditions was transferred to the pitted surface at an angle of 30°, at 0.1 mm/min, and a mmA of 110 Å^2^/macrocycle. A Brucker Dimension 3000 AFM was used in tapping and concurrent phase imaging modes under ambient conditions to image the film and pits. The Si N-type tip with a 6 nm radius (AppNano Access-NC-20) was driven at 2.66 kHz.

### Optical microscopy

The above described monolayer sample located on the pitted SiO_2_ surface was imaged with an Olympus microscope (LMPLFLN M 50× 0.50 NA) equipped with a View4 HD USB camera and its software was used for the optical imaging and contrast measurements.

### Transmission electron microscopy

TEM images were obtained with a Tecnai ST20 200 kV instrument with a LaB6 electron gun. It was not equipped with an objective aperture, preventing a measurement of electron diffraction patterns. Samples were transferred to either lacey carbon grids (SPI or Ted Pella) or ultrafine gold mesh grids (Ted Pella). The best results were obtained by first transferring a Zn-**1** film to a Ge substrate and then removing about 30% of it by a short exposure to a 90:10 mixture of CHCl_3_ and pyridine under IR monitoring. Any small amount of ZnCl_2_ co-transferred with the polymer was removed by a pyridine mixture wash. The film floated on the surface and was collected by moving an Au-6000 mesh gold TEM grid along the surface. Many of the mesh holes were completely blocked by the mass of Zn-**1**.

### Electrical conductivity

The multilayer porphene/porphite films prepared for the IR transmission and Raman experiments were used to make crude ambient temperature electrical conductivity measurements. Indium foil electrodes were pressed across the thick multilayer band of Zn-**1** by two glass plates separated by a 1 mm gap. A saturated solution of I_2_ in hexane was applied to the gap and an *I*–*V* curve was recorded from +3 to −3 V using a BioAnalytical Systems electrochemistry station. Repeated applications of the iodine solution were made until the recorded *I*–*V* curve showed no further change. Subsequent washing of the gap with neat hexanes returned the gap to a state of high resistivity exceeding the noise limit of the electrometer. When the iodine solution was reapplied, conductivity was restored.

### Density functional theory calculations

The energies of Zn-**1** (Fig. 1) and its isomers were calculated using the VASP^79,80^ code, utilizing the PBE50 functional^81,82^. A 420 eV planewave cutoff was used, layers were separated about 10 Å in the *z* direction, and both ionic positions and lattice constants were allowed to change during the optimization. To minimize Pulay stress, cell volume was kept constant throughout the optimization. Convergence thresholds of 1 × 10^−5^ and 1 × 10^−4 ^eV were used for the wavefunction and the geometry, respectively. The first Brillouin zone was sampled by a Γ-centered 8 × 8 × 1 grid in case of Zn-**1** and Zn *s*-isoporphene, while equivalent 8 × 4 × 1 and 4 × 4 × 1 grids were used for Zn *z*-isoporphene and other isomers, respectively. The GW pseudopotentials that are provided with VASP6 were used. The previously known^21,83,84^ metallic *P*4*mm* square geometry was first obtained using PBE. Reoptimization and a frequency calculation at PBE50 under symmetry constraints found the *P*4*mm* geometry to be a transition structure. Following its unstable vibration led to a rectangular *P*2*mm* minimum with an energy 3.7 kcal/macrocycle lower than the *P*4*mm* geometry. At PBE50, *P*2*mm* has an indirect band gap of 0.65 eV and an absorption maximum at 1055 nm, while *P*4*mm* is gapless. We note that the nature and relative energies of the *P*2*mm* and *P*4*mm* geometries depend strongly on the functional used.

Infrared and Raman spectra of the *P2mm* geometry were calculated with the PBE50 functional, a 420 eV plane wave cutoff, using a 6 × 6 × 1 reciprocal grid. The geometry was reoptimized with a more tight 10^−6 ^eV energy threshold, and the normal mode frequencies were determined using the finite differences approach implemented in the Phonopy code^85^. Born charges were determined from the response to finite electric fields with a magnitude of 0.002 V/Å. The infrared spectrum was generated using the Phonopy-Spectroscopy code^86^. The resonant Raman spectrum was obtained by displacing the equilibrium geometry along Raman-active normal modes and performing two TD-DFT calculations for each mode to obtain the real part of the frequency-dependent dielectric function. TD-DFT calculations utilized the Tamm-Dancoff approximation and included 8 occupied and 8 virtual states.

XPS binding energies of *P2mm* Zn-**1** and *D*_*4h*_ Zn-**2** were calculated using the FHI-aims code^87^, the PBE functional, a Γ-centered 8 × 8 × 1 reciprocal grid, and first-tier tight basis sets, at geometries optimized using PBE50. The unit cell was modified to be 50 Å long in the *z* direction, and energy corrections for charged systems up to dipole were included in the *z* direction. Hole screening was taken into account^88^.

All nonperiodic calculations were done using the Gaussian^89^ program. To estimate the footprint of a monomeric porphyrin, two molecules of **1** were locked in the same plane and the distance *d* between their ring centers was scanned from 9.5 to 12.0 Å, with an optimization performed at each step. Extrapolation from the part of the curve where the increase in energy becomes roughly linear (Supplementary Fig. 7) yielded a critical value *d*_crit_ of 10.4 Å, which corresponds to a molecular area of 108 Å^2^. The footprint of stacked Zn-**2** molecules was estimated by scanning plane-to-plane distances in a trimer in a similar manner (Supplementary Fig. 7), giving a *d*_crit_ value of 3.35 Å. These calculations were done using the PBE-D3BJ^90,91^ functional and the def2-TZVP basis set.

To check that the results are independent of the method of calculation, and in particular, of the type of basis set used, calculations were also done using the Crystal17 software^92^, employing the PBE0/pob–TZVP^93,94^ level of theory and a 6 × 6 Pack-Monkhorst net. The results were compared to VASP calculations using the PBE0 functional with a 420 eV cutoff and a 8 × 8 × 1 Gamma-centered reciprocal grid (Supplementary Table 3). In the case of Zn-**1**, Crystal17 calculations predicted a metallic conductor in agreement with previously published results^21^ and our VASP calculations. In case of the checkerboard configuration of free-base porphene, Crystal17 calculations found *P2mm* and *P4mm* minima, which were then reoptimized using VASP.

### Supplementary information

Supplementary information

## Data Availability

The data generated in this study are provided in the Main Text file and the Supplementary Information file.
