# Supplementary material for: Porphene and porphite as porphyrin analogs of graphene and graphite
Source: Nat Commun. 2023 Oct 9;14:6308. doi: 10.1038/s41467-023-41461-w (PMC10562370; doi:10.1038/s41467-023-41461-w)
Supplement: Supplementary file 1 — Supplementary information [file 41467_2023_41461_MOESM1_ESM.pdf]

Supplementary Information

Porphene and Porphite as Porphyrin Analogs of Graphene and Graphite

Thomas F. Magnera, Paul I. Dron, Jared P. Bozzone, Milena Jovanovic, Igor Rončević, Edward Tortorici, Wei Bu, Elisa M. Miller, Charles T. Rogers, and Josef Michl

Correspondence to: Josef.Michl@colorado.edu

TABLE OF CONTENTS

	Page
Supplementary Figure 1	2
Supplementary Figure 2	3
Supplementary Figure 3	4
Supplementary Figure 4	5
Supplementary Figure 5	6
Supplementary Figure 6	7
Supplementary Figure 7	8
Supplementary Figure 8	9
Supplementary Figure 9	10
Supplementary Figure 10	11
Supplementary Figure 11	12
Supplementary Figure 12	13
Supplementary Figure 13	14
Supplementary Table 1	15
Supplementary Table 2	16
Supplementary Table 3	18
Supplementary Table 4	19
Supplementary Note 1	20
Supplementary Note 2	21
Supplementary Note 3	27
Supplementary Note 4	30
Supplementary Note 5	31
Calculated Optimized Geometries	34
Supplementary References	40

SUPPLEMENTARY FIGURES

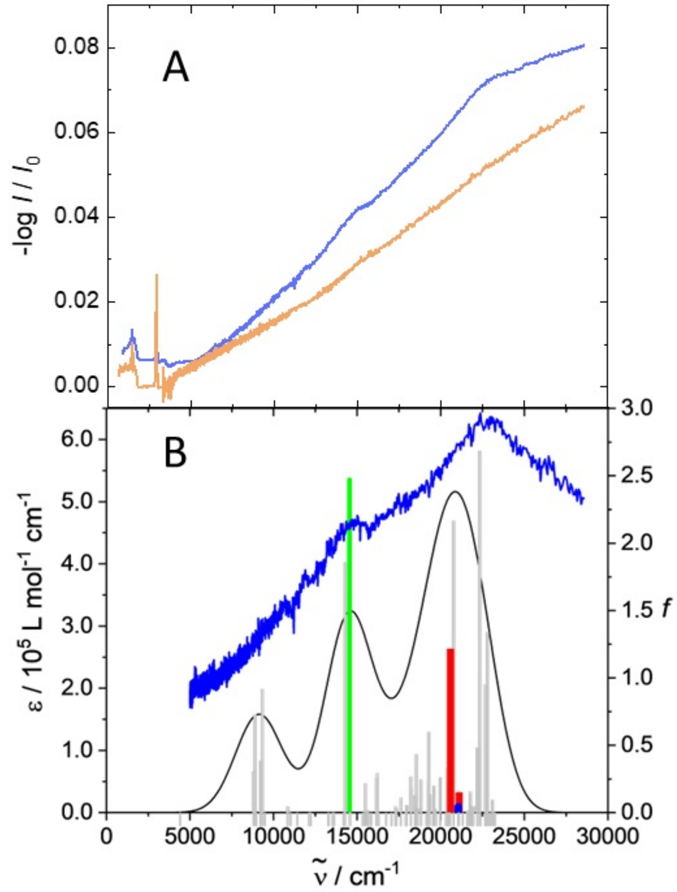

Supplementary Figure 1. (A) UV-vis-NIR-IR absorption spectra of Zn-**1** prepared under standard (blue) and exhaustive (orange) oxidation conditions. (B) Spectrum of defects in Zn-**1** (blue curve) from difference of spectra shown in A, arbitrarily scaled for ease of comparison, and TDDFT/PBE50 calculated spectrum of a nine-macrocycle model (Supplementary Figure 11) of the defect (black curve; obtained by broadening lines in the bar spectrum into 0.2 eV width Gaussians and summing over all transitions). Bars indicate energies and oscillator strengths *f* of individual transitions (lines pointing down have zero or negligible intensity). Transitions shown in gray and green are localized outside of the twisted macrocycle. Green, *y*-polarized; red, *x*-polarized; blue, approx. *z*-polarized. Transition densities for the colored bars are shown in Supplementary Figure 12ABCD, ordered *y*, *x*, *z*, *x* in increasing transition energy.

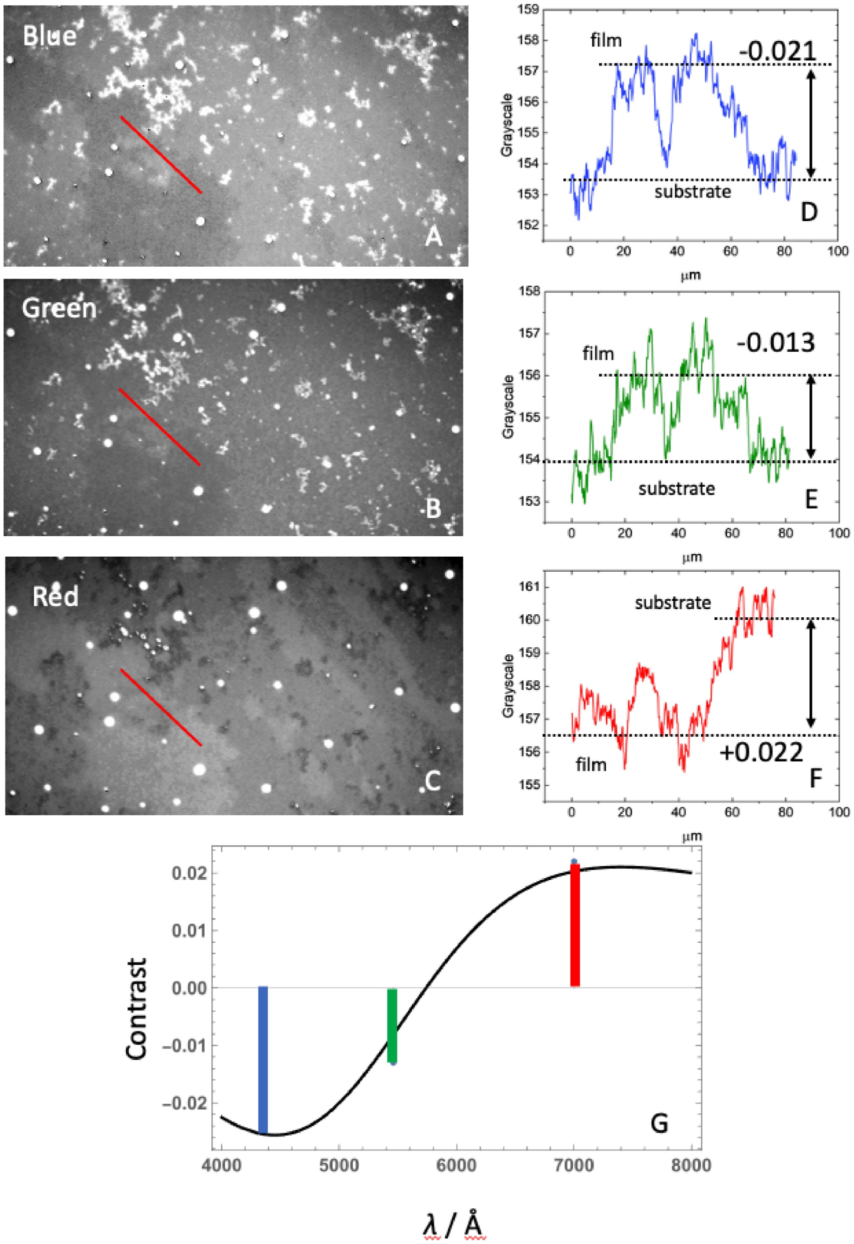

Supplementary Figure 2. (A-C) RGB optical microscopy image of Zn-**1** on pitted SiO_2 (120 nm thick on Si, Main Text Figure 6a) was decomposed into individual red (A), green (B) and blue (C) images. (D-F) Contrast profiles along the red lines are shown in A-C. The contrast was enhanced for A-C to improve the presentation, but the grayscale values in D-F are absolute. (G) Calculated contrast (black curve) for the refractive index of **1** (n,k) = $(2.55,k)$, where k is calculated from the absorptivity of a 20 nm thick film (Supplementary Figure 1). The blue, green and red vertical bars mark the observed contrast at the three wavelengths.

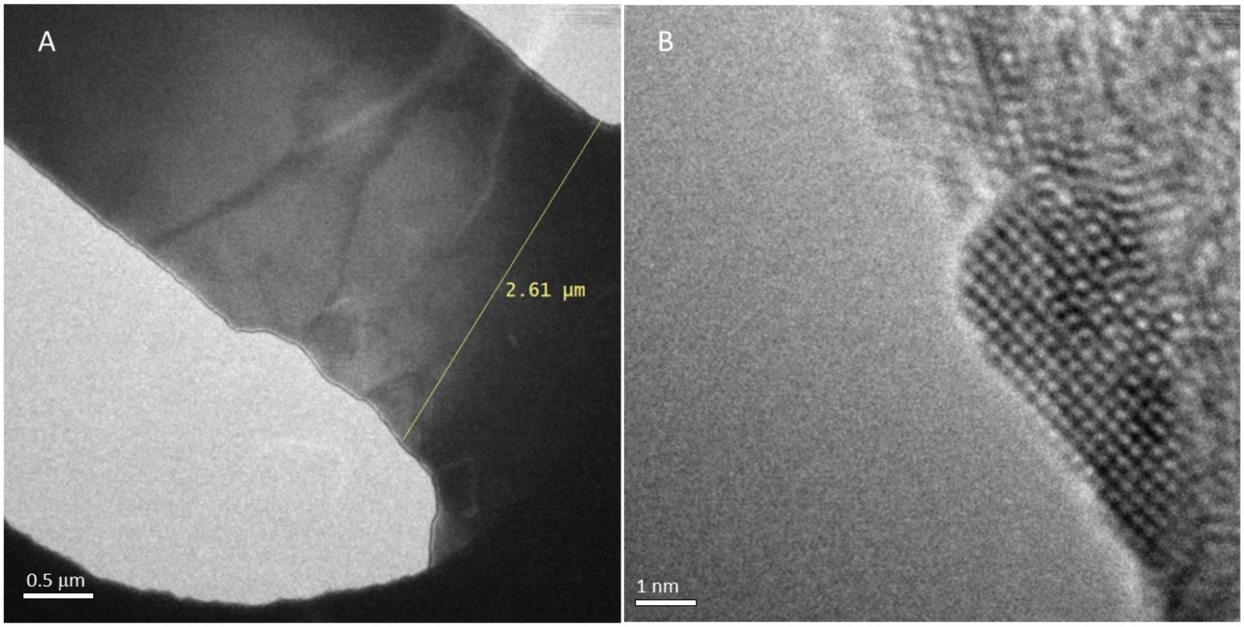

Supplementary Figure 3. TEM images of Zn-**1** porphite prepared under standard conditions. (A) A flake spanning a 6 µm circular hole in an Au mesh. (B) A thin flap protruding from an edge of the flake shown in (A).

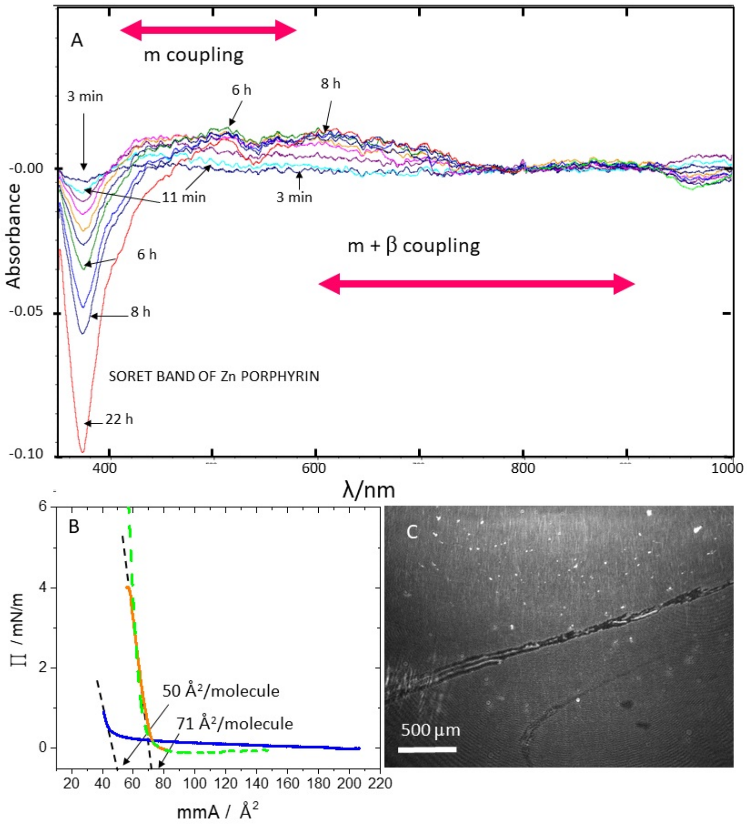

Supplementary Figure 4. (A) UV-vis difference absorption spectra at air/water interface taken in successive intervals during oxidative polymerization of Zn-**2** to **1** with K_2IrCl_6 (initial spectrum is subtracted). (B) Isotherm of Zn-**2** on neat water (blue), aqueous 167 μM K_2IrCl_6 (orange), and aqueous 167 μM K_2IrCl_6 followed by treatment with 200 μM NaI and then 0.1 M $ZnCl_2$ (dashed green). Dashed black lines extrapolated to the baseline reveal the mmA of condensed material. (C) BAM of Zn-**1** on a subphase of 0.1 M $ZnCl_2$ at pH 3.

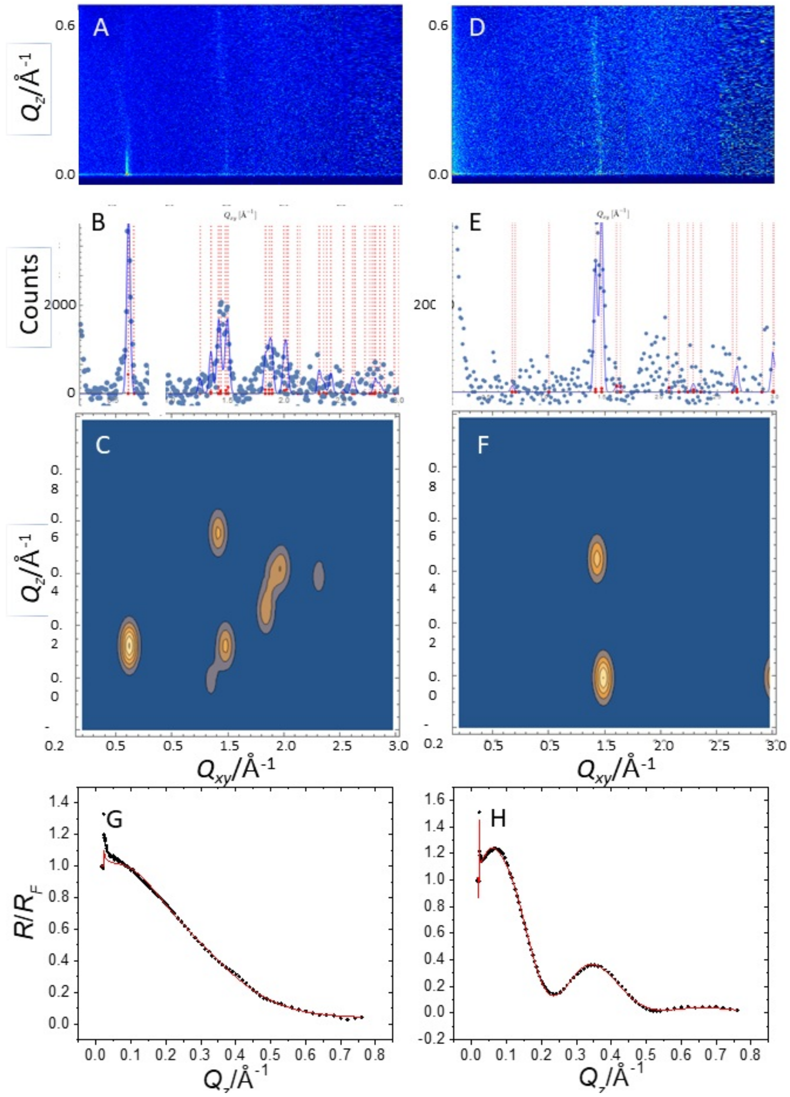

Supplementary Figure 5. (A) GIXD of a Langmuir layer of Zn-**2** before polymerization. (B) Intensity of A integrated in the Q_z direction (blue circles), the fitted curve (blue, solid), and positions of indexed peaks (red, dashed). (C) Fitted intensity for (Q_{xy},Q_z). (D) GIXD of a Langmuir layer of **1** after polymerization. (E) Intensity of D integrated in the Q_z direction (blue circles), the fitted curve (blue, solid), and positions of indexed peaks (red, dashed). (F) Fitted intensity for (Q_{xy},Q_z). The peak widths in (C) and (F) are widened to enhance their visibility. (G, H) Normalized reflectivity curves (black) and slab model fits (red) for Langmuir layers of Zn-**2** before (G) and **1** after (H) polymerization.

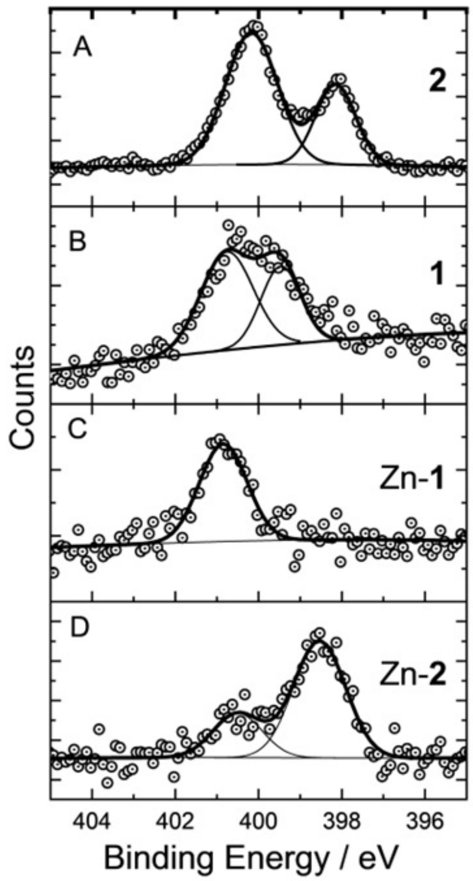

Supplementary Figure 6. The N(1*s*) binding energy region in XPS of films on ITO. Cast films of **2** and Zn-**2**, and LB films of **1** and Zn-**1**. Solid lines show the fitted curves, baseline, and component peaks used in the total fit.

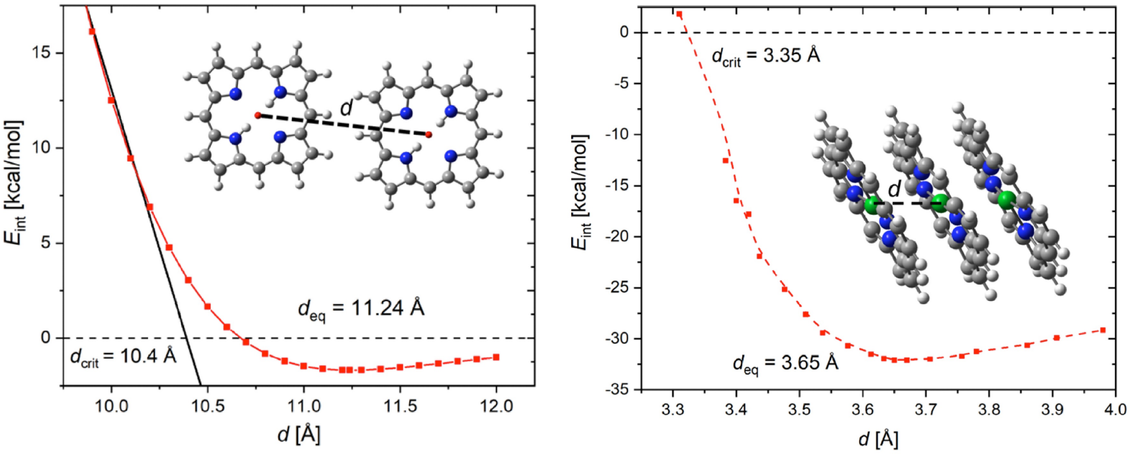

Supplementary Figure 7. PBE-D3BJ/def2-TZVP energies relative to energy at infinite separation, providing equilibrium (d_{eq}) and critical (d_{crit}) distances. Left: center-to-center distance d between two coplanar molecules of **2**. Right: Horizontal distances d between stacked molecules of Zn-**2** in a trimer.

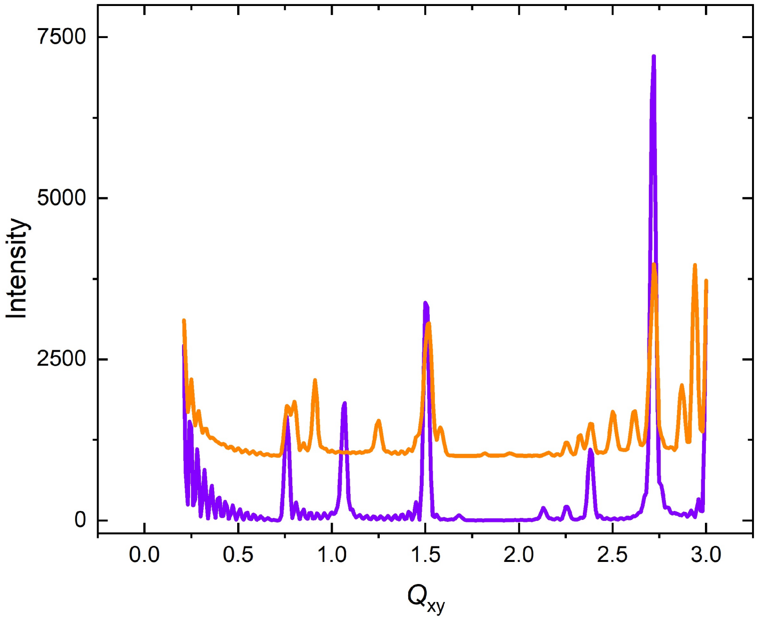

Supplementary Figure 8. The expected GIXD patterns of a monolayer of porphene **1** (orange) and of *s*-isoporphene (see Main Text Figure 1e, purple) are distinctly different.

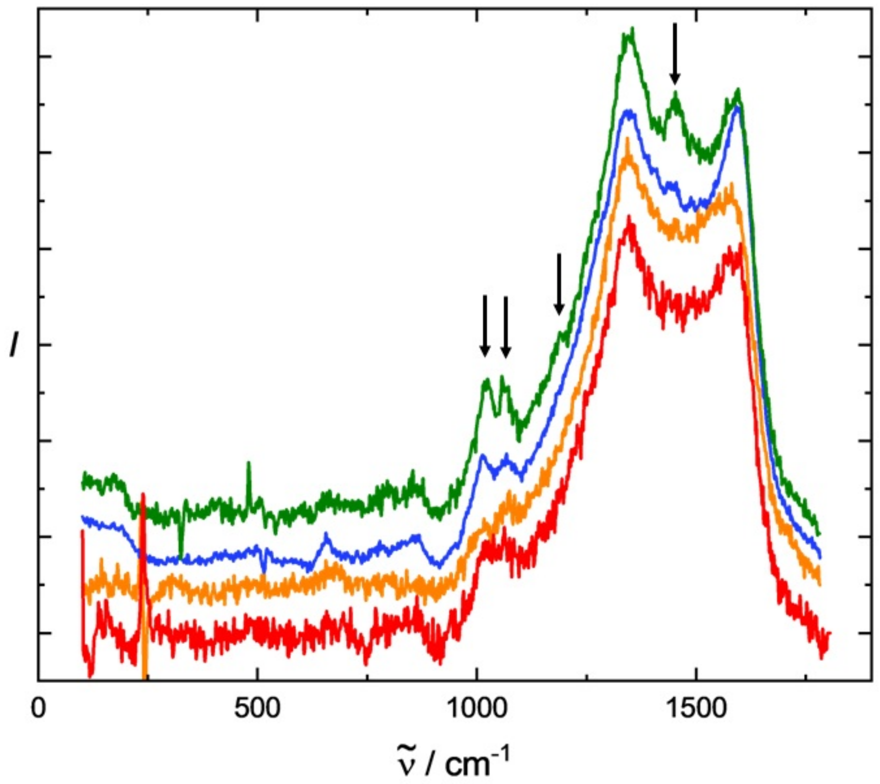

Supplementary Figure 9. Disappearance of Raman bands of defects in Zn-**1** after transfer to BaF_2 (marked with arrows) with increasing reaction time and K_2IrCl_6 concentration: (green) 12 h, 35 μM; (blue) 72 h, 35 μM; (orange) 22 h, 350 μM; (red) 120 h, 210 μM.

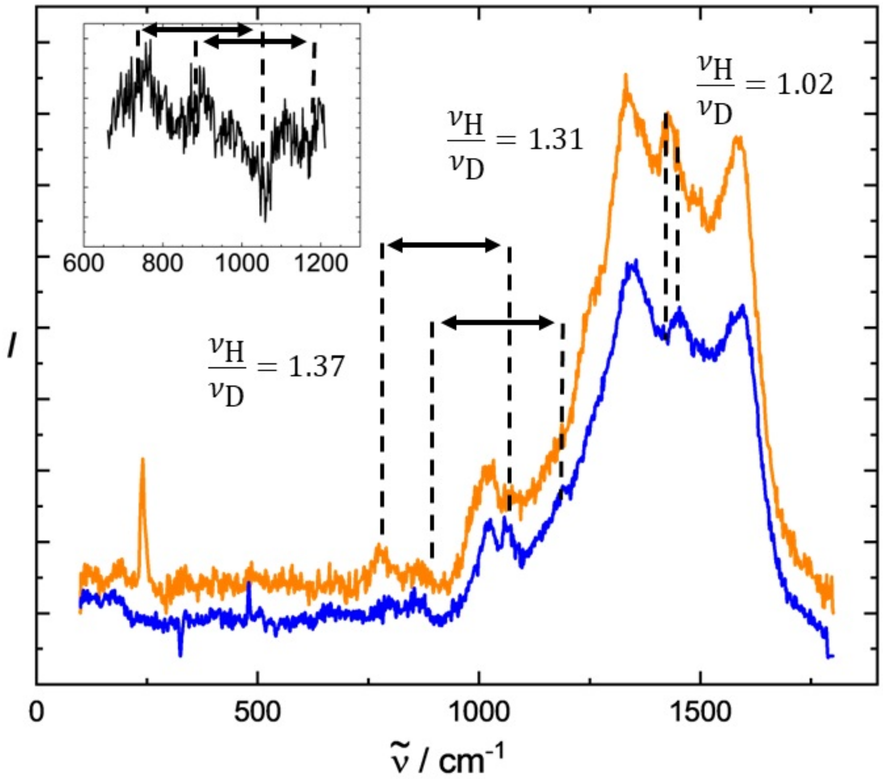

Supplementary Figure 10. Raman spectra of Zn-**1** transferred to CaF_2 after standard polymerization of **1**-h_{12} (blue) and **1**-d_{12} (orange). Inset: Difference spectrum shows positive Zn-**1**-d_{12} and negative Zn-**1**-h_{12} bands.

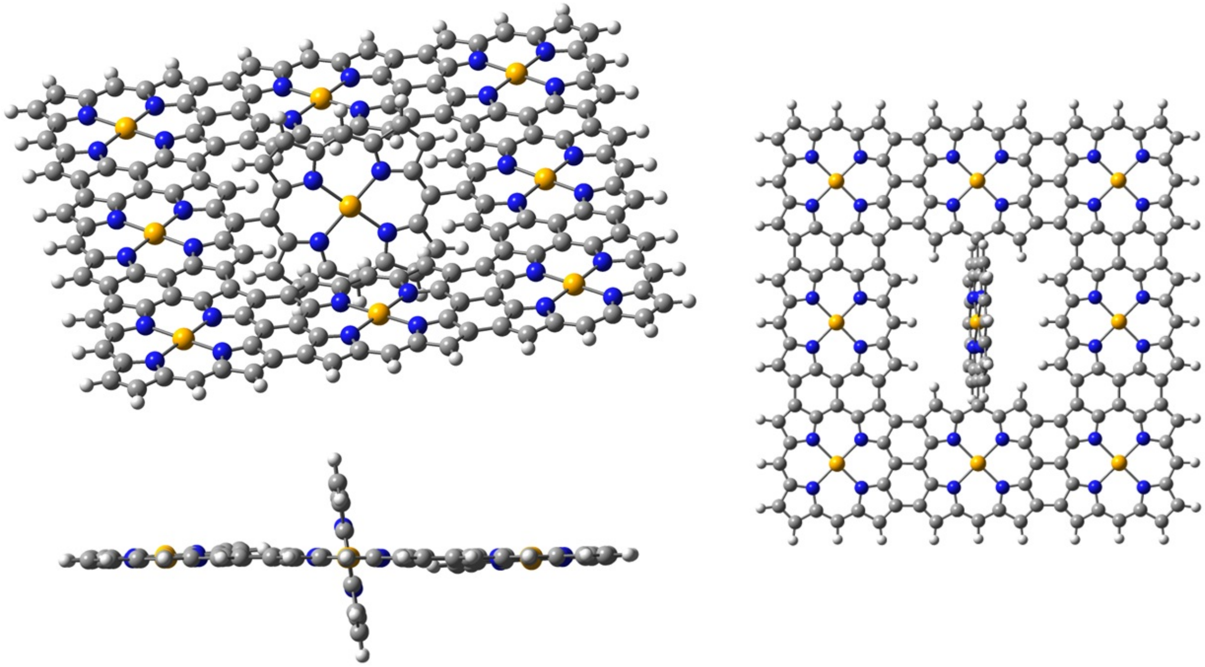

Supplementary Figure 11. DFT/PBE50 optimized geometry of a simple model of a defect in Zn-**1**.

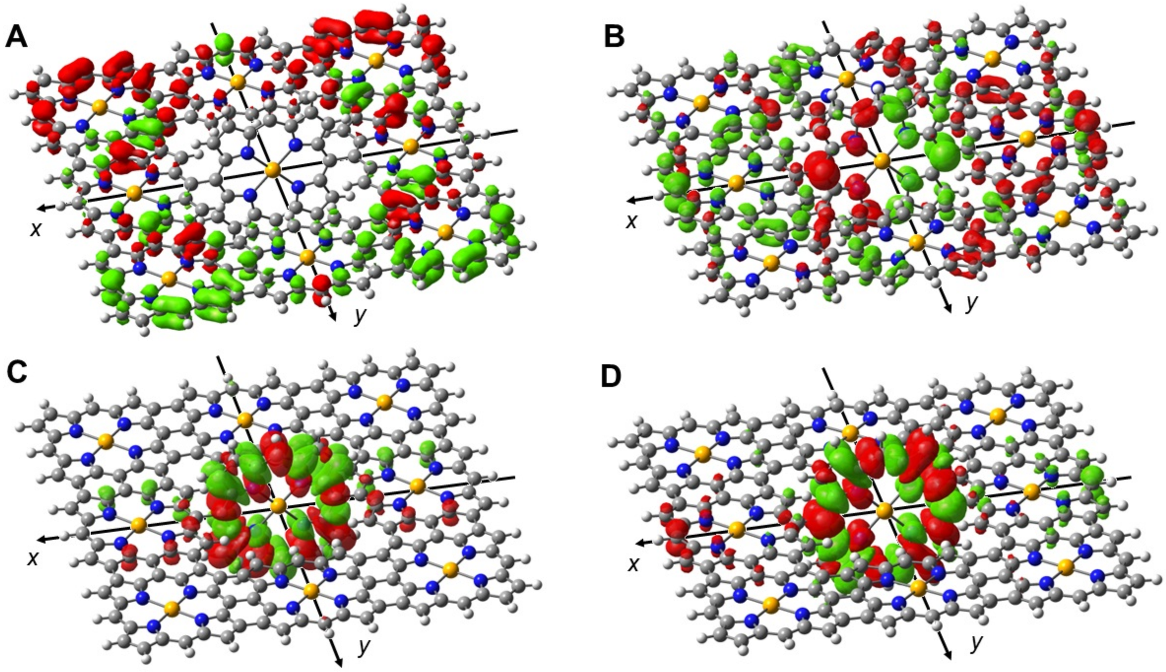

Supplementary Figure 12. TDDFT/PBE50 transition densities for excitations marked by colored bars in Supplementary Figure 1. Panels A-D correspond to green, red, blue, and red bars in the order of increasing transition energy, respectively.

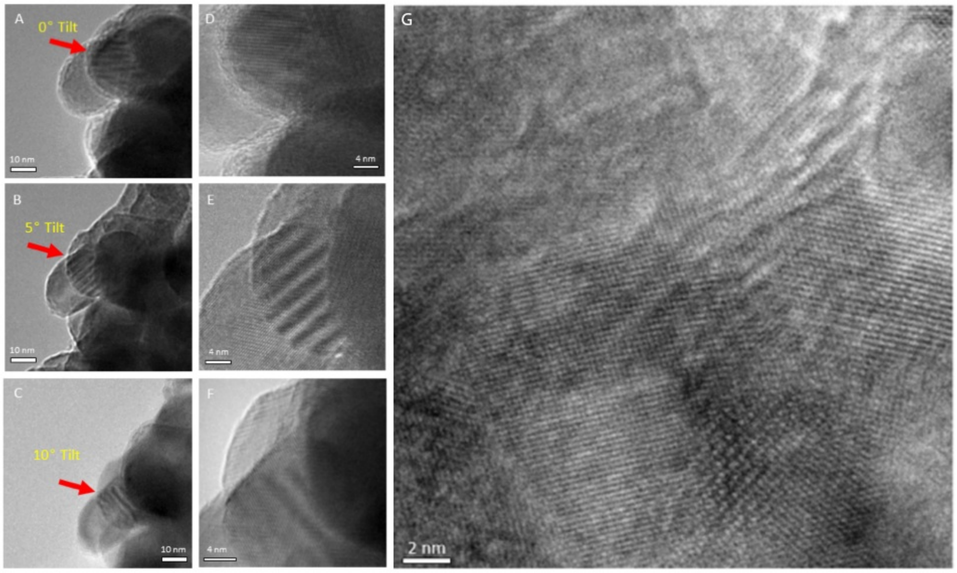

Supplementary Figure 13. (A-C) TEM images of the same section of a Zn-**1** porphite slab viewed at three different angles. (D-F) Enlarged sections of (A-C). (G) TEM image of an interior section of Zn-**1** porphite shown in panel A with many overlapping stacked layers of Zn-**1** and moiré patterns.

SUPPLEMENTARY TABLES

Supplementary Table 1. Diffraction peaks observed for a bilayer of Zn-**2** on water before and after oxidative polymerization to a layer of **1** combined with a layer of anions, indexed for the porphene primitive unit cell (Roman) and the $IrCl_6^{-2}$ unit cell (italic).

**before polymerization (Zn-2)**						
h	*k*	*l*	$(Q_{xy},Q_z)_{hkl}$ (obsd)	$(Q_{xy},Q_z)_{hkl}$ (calcd)	I_{hkl} (obsd)	I_{hkl} (calcd)
0	-1	0	(0.634,0.1)±0.1	(0.622,0.09)±0.1	3600±10%	(42342)[a] 42342[b]
-1	2	0	(1.43,0.55)	(1.42,0.56)	1446	(8000) 1261
2	-1	1	(1.50,0.1)	(1.49,0.09)	1585	(24,821) 1261
**after polymerization (1) Model A**						
6	*-1*	*1*	*(1.45,0.47)*	*(1.44,0.47)*	*3400*	*3353*
2	0	0	(1.50,0.00)	(1.50,0.00)	3542	2968
**after polymerization (1) Model B**						
-3	*3*	*-1*	*(1.45,0.47)*	*(1.42,0.47)*	*3400*	*2301*
2	0	0	(1.50,0.00)	(1.50,0.00)	3542	3559
**after polymerization (1) Model C**						
0	-2	0	(1.45,0.47)	(1.44,0.53)	3400	(2446) 2995
-4	0	-1	(1.50,0.00)	(1.50,0.00)	3542	(3642) 2850

[a] Intrinsic intensity. [b] Intrinsic intensity scaled and multiplied by the Deybe-Waller factor.

Supplementary Table 2. X-ray reflectivity of a Langmuir layer. Top, before polymerization; center (Model C) and bottom (Model A), after polymerization. [a]

Slab	L_{calcd}/Å	L_{fit}/Å	n_e [b]	$n_{e,\ fit}$	$\rho/(e^-/Å^3)$	σ_{fit}/Å
He			0		0	8.22±0.3[c]
Zn-**2** ($N_4C_{20}Zn$)	4.0	4.04±0.05[c]	190	171	0.695±0.1[c]	2.50
interlayer	1.0	0.0	0		0	7.70
Zn-**2** ($N_4C_{20}Zn$)	4.0	4.00	190	221	0.903	6.95
trans. zone		3.08	0	15	0.082	5.93
subphase	∞				0.333	
-------------------	----------	-----------	------------	--------	-----------------	--------------
He			0			4.08 [2.52]
**1** ($N_4C_{20}H_2$)	2 [2]	2.17 [2]	150 [150]	140	0.90 [0]	17.6 [4.11]
hydrophobic layer	1.5 [1.5]	2.01	0	0	0.0	1.91 [3.0]
$IrCl_{6\text{-}2}$	8 [-]	4.71 [-]	179[e] [-]	150 [-]	0.44 [-]	2.60 [-]
trans. zone	[-]	2.86 [-]	[-]	121 [-]	0.59 [-]	2.0 [-]
subphase	0				0.333 [0.333]	
-------------------	----------	-----------	------------	--------	-----------------	--------------
He			0			
**1** ($N_4C_{20}H_2$)	2 [2]		150 [150]		0.90 [0]	
hydrophobic layer	1.5 [1.5]		0		0.0	
$IrCl_{6\text{-}2}$	8 [-]		179[e] [-]		0.44 [-]	
trans. zone	[-]		[-]		0.59 [-]	
subphase	0				0.333 [0.333]	
---------------	----------	-----------	------------	------	----------------	--------------
He			0			5.54
**1** ($N_4C_{20}H_2$)	2.0 [-]	2.0	150 [-]	151	2.08[d] [-]	3.89 [-]
interlayer	3.3 [-]	3.3	0 [-]	3	0.03 [-]	5.45 [-]
**1** ($N_4C_{20}H_2$)	2.0 [-]	2.06	150 [-]	153	2.08 [-]	8.37 [-]
trans. zone	8.0 [-]	8.21	279[e] [-]	119	0.40 [-]	4.2 [-]
subphase	∞				0.333 0.333	

[a] Our DFT calculations show that electron density in Zn-**2** projected into the direction perpendicular to the porphyrin ring is fitted well by a Gaussian with FWHM of about 1 Å from which we obtain 2 Å for the total thickness L of the monolayer slab, which is doubled for the tilted bilayer of Zn-**2**. [b] Number of electrons. Electron density ρ for a 2 Å layer of Zn-**2** (190 e^-) at the mmA of the

measurement is estimated from n_e / (L × mmA) = 190 e^-/(2 Å × 72 Å^2) = 1.5 e^-/Å^3 and for a 2 Å layer of **1** (150 e^-), ρ = 1.0 e^-/Å^3. The transition zone gap distance for **1** was estimated as twice the radius of the $IrCl_6^{-2}$ anion ($r_{\text{Ir-Cl}}$ = 2.3 + van der Waals radius of Cl, 1.75 Å = 4.04 Å). Expected density for one anion per monomer unit is 179 e^- + H_2O e^-/ (72.5 Å^2 × 8.08 Å) = (179+103.1)/585.8, where the number of electrons from H_2O is found from ρ_{H2O} (V_{total} - V_{IrCl6}) = 0.3333(585.8 - 276.2) = 103.1 and ρ_{IrCl6} = 0.48 e^-/Å^3, for an occupancy of 2 is 0.63 e^-/Å^3 and for 0.5 is 0.41 e^-/Å^3. [c] Std. error. [d] Adjusted for mmA of bilayer. [e] Electrons expected for one $IrCl_6^{-2}$ with the remaining volume backfilled with H_2O. [f] Numbers on the left: with $IrCl_6^{-2}$ present; numbers in brackets: with $IrCl_6^{-2}$ absent; [-]: not applicable. [g] Fitting did not converge for this model.

Supplementary Table 3. Computed relative energies (E_{rel}), band gaps (E_g), and lattice constants (*a*, *b*) for the rectangular (*P*2*mm*) and square (*P*4*mm*) valence isomers of checkerboard free-base porphene, obtained using the PBE0 functional.

Code<br>Level of theory	Crystal17<br>PBE0/pob-TZVP		VASP<br>PBE0/420 eV cutoff	
Geometry	*P*2*mm*	*P*4*mm*	*P*2*mm*	*P*4*mm*
E_{rel} (kcal/mol.macrocycle)	0.0	1.45	0.0	1.22
E_g (eV)	0.14	0	0.16	0
a (Å)	16.84	16.73	16.85	16.75
b (Å)	16.64	16.73	16.66	16.75

Supplementary Table 4. Reference Vibrational Frequencies of Zn-**2**-h_{12} and Zn-**2**-d_{12}.[a]

ν_H/cm^{-1}	ν_D/cm^{-1}	ν_H/ν_D (obsd.)	ν_H/ν_D (calcd.)	Mode
1020	1020	1.0	1.01	pyrrole ring
1063	774	1.37	1.39	δC_β-H
1190	902	1.31	1.31	δC_m-H
1454	1428	1.02	1.01	C_m-C_α
3062				νC_m-H
3097				νC_β-H

[a] Li, X. Y. and Zgierski, M. Z. Porphine force field: in-plane normal modes of free-base porphine: Comparison with metalloporphines and structural implications. *J. Phys. Chem.* **95**, 4268-4287 (1991).

SUPPLEMENTARY NOTES

Supplementary Note 1

LB and BAM. Isotherms of Langmuir films of porphyrins as a class are known to have poor behavior with early reports[1,2] suggesting that this was due to an easy conversion from a flat-lying to an on-edge configuration.[3]

A Langmuir layer of Zn-**2** spread from a 2.8 μM benzene solution at room temperature onto water was compressed from over 200 Å^2/molecule to a surface pressure (Π) of ~1 mN/m at 40 Å^2/molecule (Supplementary Figure 4B). Extrapolation to the baseline yielded a mmA of 50 Å^2/macrocycle, much too small for Zn-**2** lying flat on the surface in a monolayer, for which molecular modeling suggests ~108 Å^2/macrocycle (Supplementary Figure 7). The value is slightly too small even for the Zn-**2** molecules lying perfectly flat in a bilayer, whose observed footprint would then be 108 Å^2/macrocycle in each layer, but it would be compatible with a bilayer in which the molecules are tilted by about 22°.

As the film is compressed the isotherm starts to deviate from the baseline near a mmA of 190 Å^2/macrocycle and slightly rises by a fraction of a mN/m until it approaches the extrapolation point, after which it rises rapidly. Close inspection of the isotherm reveals numerous small upward steps and drops in Π, indicative of the non-ideal behavior considered typical for a Langmuir film of a porphyrin[1] and suggestive of a local bilayer or possibly even multilayer formation.

The orange line in Supplementary Figure 4B was obtained by first spreading an identical amount of Zn-**2** on the surface of a 167 μM aqueous solution of K_2IrCl_6, then cycling it for the next 6 h between barrier positions corresponding to 90 and 71 Å^2/macrocycle, and only then obtaining the isotherm for the resulting polymer **1**. It has an extrapolated mmA of 71 Å^2/molecule. In the GIXD section below it will be seen that this corresponds almost exactly to a full coverage of the surface with a monolayer of **1**, whose unit cell has a (8.4 ± 0.1) × (8.4 ± 0.1) Å^2 footprint. However, this isotherm is very poorly reproducible and the polymerization of Zn-**2** leads to mmA/macrocycle values that range from 65 to 110 Å^2 and can exhibit considerable hysteresis. This behavior is perhaps not surprising, given that the surface presumably contains a heterogeneous collection of flakes of **1** of unknown size and shape fitting together randomly, along with an ill-defined collection of unreacted Zn-**2** and small oligomers, plus dirt that has fallen onto the surface over the hours of the synthesis.

The dashed green isotherm in Supplementary Figure 4B is for a Langmuir film of Zn-**1** synthesized from **1** by oxidative polymerization of Zn-**2** spread on an K_2IrCl_6 subphase solution as described just above and converted to Zn-**1** by first adding an excess of NaI to the subphase and then making it ~0.1 M in $ZnCl_2$ and waiting for 24 h. The similarity of the isotherms of the electroneutral Zn-**1** and the positively charged **1** with its layer of counterions underneath shows that heavy doping of the polymer with positive charges has minimal effect on its overall structure. Theory indeed suggests that neither charging by electron removal or by protonation of a third nitrogen in the macrocycle, nor insertion of a metal into the macrocycles changes the shape of the porphyrin ring in **1** significantly. The footprint of a macrocycle within the two-dimensional polymer was calculated for **1** (70.9 Å^2), the polyradical polycation of **1** with every macrocycle positively charged (71.1 Å^2), the polycation of **1** formed by protonation of one nitrogen in every macrocycle (71.3 Å^2), for Zn-**1** (70.8 Å^2), and for the Zn-**1** polyradical polycation with every macrocycle positively charged (70.9 Å^2). Similarly, DFT calculations of the center-to-center distances between macrocycles in the zinc salt Zn-

1 (8.41 Å) and in various forms of the free base **1** yield virtually identical results for all: 8.42 Å in the windowpane (all NH bonds parallel), 8.41 and 8.44 Å in the striped (NH bond directions alternating in parallel stripes), and 8.43 Å in the checkerboard (NH bond directions different in the white and black fields) tautomer.

BAM images (e.g., Supplementary Figure 4C) show a gray area with indistinct long lines believed to be due to cracks between polymer flakes, and a small number of light points, most likely nanocrystals of unreacted Zn-**2**. Before polymerization, random motions of these points, due to factors such as air drafts, are uncorrelated, while after polymerization, they are correlated and all move together, demonstrating mechanical rigidity of very large flakes of the surface layer.

Supplementary Note 2

GIXD. Supplementary Figure 5AD shows the in situ interfacial GIXD scattering peaks (Q_{xy},Q_z) for unpolymerized (Zn-**2**) and polymerized (**1**) compressed Langmuir films, the former floating on neat water (1.0 mN/m, 60 Å^2/macrocycle) and the latter on a 167 μM aqueous solution of K_2IrCl_6 (4.0 mN/m, 72 Å^2/macrocycle).

Unpolymerized Film (Zn-**2**, Supplementary Figure 5B). The integrated scattering intensity has two major on-axis peaks at 0.634 and 1.50 Å^{-1}, with an estimated intensity close to Q_z = ~0.1 Å^{-1} (from fits to the Bragg rod profile), one significant off-axis peak at 1.43 Å^{-1} (Q_z = 0.55 Å^{-1}), and an unresolved group near 1.86 Å^{-1} with unassignable off-axis contributions masked by off-axis background scattering. The original data contain a very broadly peaked background scattering intensity due to the subphase, and this has been removed from all observed spectra shown. The rising scattering intensity at the lowest Q_{xy} values is due a contribution from the primary beam. A slightly oblique θ = 84.4 ° primitive 2-D unit cell (9.34 × 10.08 Å, 84.4°, *P1*) with nearly flat-lying porphyrin rings tilted at 24.8 ° from the surface in the direction of the short side of the unit cell is used to assign Miller indices to the observed peaks (Supplementary Table 1). In Supplementary Figure 5B, red dotted vertical lines indicate the indexed positions of diffraction peaks and a blue solid line is a fit to the integrated intensity, which used the atomic positions found in the crystal of a dimer,[4] holding their relative coordinates fixed. A dearth of scattering peaks in a GIXD pattern of a many atom system, as observed here, usually implies systematic absences arising from a non-primitive unit cell. In our case, it was found that the presence of a second layer 2-D lattice, with a small interlayer spacing, pushed the scattering above Q_z> 0.66 Å^{-1} and outside the range of experimental detection for many of the indexed peaks, making the observed pattern sparse. The final intensity fits were done with an upper-layer offset (S_a,S_b) = (0.5,0.08) with respect to the fractional distance along the short and long edges of the unit cell, respectively, and an interlayer spacing of 3.5 Å. The fits are reasonable, but a mmA of 46.5 Å^2/macrocycle (93.0 Å^2/macrocycle in each layer) calculated from the unit cell is smaller than the 60 Å^2/macrocycle dictated by the barrier position and in reasonable agreement with the 50 Å^2 mmA/macrocycle found from the film isotherm. Finally, it was found that a small twist of 14.4° improves the fit and when combined with the tilt follows from the slightly smaller dimensions of the unit cell compared to those expected from calculation for **2** (10.4 × 10.4 Å, *P2*). The intensity fit requires that the compound be fully metallated. The overall decrease in intensity due to surface and vibrational motion is accounted for using a Debye-Waller factor[5] of 0.63 Å. The average fitted FWHM is 0.018 Å and corresponds to a domain size of 37.8 nm as calculated from the Scherrer formula[5] $\ell = 0.88 \times 2\pi/Q^{Corr}_{xy}$ with standard correction. The poorly fitted weak

intensity at Q_{xy} = 0.81 Å^{-1} is attributed to domains of a secondary surface structure embedded in the film. Primitive and non-primitive unit cells with the porphyrins tilted and twisted on their edge were tested and failed to reproduce the observed intensity pattern. At the surface pressures at which the GIXD was measured it is unlikely that Zn-**2** would be forced on its edge in crystalline domains that were reported previously[2,6] for other porphyrins.

Polymerized Film (**1**, Supplementary Figure 5DE). The GIXD has only two significant peaks at 1.45 and 1.5 Å^{-1}. The absence of intensity for [10*l*] and [01*l*] peaks expected at Q_{xy} = 7.22 and 7.53 Å^{-1} requires the structure to be the completely demetallated **1** (see below). The first of the observed peaks is off-axis at Q_z = 0.47 Å^{-1}. A rectangular primitive 2-D unit cell (θ = 90°, 8.34× 8.70 Å, *P2mm*), whose 73.0 Å^2 mmA/macrocycle is shrunk relative to that of the film of unpolymerized Zn-**2**, was used to index the scattering pattern (Supplementary Figure 5E, red dotted vertical lines). Supplementary Table 1 summarizes the peak positions and their assigned Miller indices.

In spite of the dearth of GIXD peaks observed, an examination of many possible structures identified only three unit cells that provide a good fit (predicted peak intensities at higher Q_{xy} were attenuated by a Debye-Waller factor[5] of 0.57 Å).

Model A is a superlattice of monolayer **1** with a *P*4*mm* 2-D unit cell above a second larger *P*4*mm* lattice containing two rotationally disordered $IrCl_6^{-2}$ ions whose lattice vectors are twisted out of alignment with those of **1**. Model B is the same as model A, except that the unit cell is not body-centered and the interlayer distance is doubled. Model C, which provides a slightly worse fit, is a bilayer unit cell of **1** containing four porphyrin macrocycles.

A. In the first model, the upper layer is a perfectly regular sheet of **1** whose unit cell (8.4 × 8.4 Å^2, 90°, *P*4*mm*) contains a single macrocycle and determines the positions of a set of scattering peaks in the GIXD. The oxidant in the subphases charges the monolayer positively and forms ion pairs with it by depositing $IrCl_6^{-2}$ ions beneath its surface in a second lattice with a different unit cell (26.5 × 26.5 Å, 90 °, *P*4*mm*), which determines the position of a second set of GIXD peaks, independent of the first. The relative intensities of the peaks from the two lattices are coupled through the scattering interference which becomes strong when their reflection planes are aligned and commensurate and leads to off-axis scattering since there is now a well-defined thickness to the system leading to principal maxima along the $\boldsymbol{Q}_z$ direction. The best fits are found if the anion lattice is non-primitive and 2-D body centered with one corner offset relative to the polymer unit cell by (0.5,0) and twisted by 18.5° so that the second corner along an edge sits beneath the macrocycle that is one to the left and three up from the first (Main Text Figure 2). The interlayer separation is 3.6 Å and the resulting bilayer structure is chiral.

B. This variation on Model A has a 18.7 × 18.7 Å^2 primitive 2-D unit cell and a 7.2 Å interlayer distance. The change of the unit cell size with the retention of the same bilayer offset requires a doubling of the interlayer spacing.

C. In the bilayer model with a non-primitive 2-D unit cell (θ = 90 °,16.68 × 8.70 Å, *P*2*mm*), an interlayer spacing of 3.4 Å, and a mmA of 36.3 Å^2/macrocycle, the upper layer is offset by (S_a,S_b) = (0.25,0.13), similarly as in the best fit for the unpolymerized Zn-**2**. If the layers of **1** were assumed to be planar, the significant asymmetry of this *P*2*mm* 2-D orthogonal unit cell would disagree with the published[7] calculations for free-base **1** with a checkerboard arrangement of NH-HN diagonals, calculated to be the most stable tautomer, and with our own calculations for monolayers through tetralayers and an infinite stack (porphite). All calculations predict planar sheets and symmetry very

close to *P4mm*, with squares only slightly distorted into diamonds (for the windowpane, stripe, and checkerboard tautomers, the calculated NH-N-NH.angles are 88.3, 88.9, and 88.6°, and the N-NH-N angles are 91.7, 91.1, and 91.4°, respectively).

The disagreement can be alleviated if Model C is modified by choosing the rectangular 2 × 1 non-primitive unit cell given above in which **1** is not flat but has a regularly non-planar geometry in which the central eight-membered ring is lifted slightly above the average plane, making the length of long edge projected into the plane 16.68 Å, rather than twice 8.70 Å . The waviness contracts the distance between macrocycle centers by 0.3 Å in one relative to the other edge of the unit cell. A disagreement with theory remains, since computations predict a planar structure with a square unit cell edge of 8.4 Å and not 8.70 Å.

All three models require a high concentration of $IrCl_6^{-2}$ anions just below the surface. A precedent[8] exists for a concentration of $PtCl_6^{-2}$ dianions in the Stern layer beneath a positively charged Langmuir monolayer that exceeds its diffuse layer value. In this case the sublayer dianion concentration was so high relative to the bulk that it surpassed the value necessary for charge neutrality in the Langmuir layer by a factor of 1.5, and extra countercations were distributed in the lower diffuse layer. Divalent cationic 1 × 2 and 2 × 2 superlattices beneath Langmuir films of long-chain alkylamines have also been studied[9] and the interactions were strong enough to buckle the Langmuir film with a displacement of 2.5 Å. This is comparable to the magnitude needed to convert **1** from *P4mm* to *P2mm*, with one side of the primitive cell shortened by 0.3 Å due to buckling.

Comparison with the LB isotherms, XR data, TEM, and results of DFT calculations favors model A. The disadvantage of Model B is that a doubled interlayer thickness disagrees with the XR results (see below). The advantages of A over C are: (i) It yields a 71 Å^2 mmA/macrocycle in much better agreement with the 65 - 110 Å^2 isotherm value. Although the observed value is poorly reproducible and widely scattered, in many repeated experiments it never came close to the 36.3 Å^2/macrocycle that Model C demands. To accommodate the discrepancy one would have to assume that in this particular measurement at least half of the surface is always covered by random debris generated in the polymerization process and/or deposited from the atmosphere. (ii) It has the correct off-axis scattering Q_z intensity while keeping **1** as a monolayer. (iii) It has sufficient symmetry to produce systematic absences in a multiatom structure. (iv) A Gaussian-fitted composite set of Bragg rod profiles at Q_{xy}=1.44 Å^{-1} gives the overall thickness of the layer of electron scattering density above the surface as ~10 Å. Taking this thickness to be equal to the (3.6 Å) interlayer spacing deduced from the GIXD pattern plus twice the van der Waals radius, we expect ~11.6 Å from the superlattice Model A and ~7.6 Å from the bilayer model C, and the former is again favored (a van der Waals radius of 4.04 Å for $IrCl_6^{-2}$ has been used[10]). (v) The Zn-**1** bilayer structure of a transferred film revealed in the TEM images (Main Text Figure 7 and Supplementary Figures 3 and 13) has a (0.5,0.5) offset that is at variance with the offset predicted by model C.

All attempts to fit the diffraction pattern to an isoporphene structure failed. Supplementary Figure 8 exemplifies the clear difference between the isomers. Considering a superlattice of the kind shown for porphene in Main Text Figure 2 led to no improvement.

GIXD Intensity Fitting. We have not found a published analytical treatment of the GIXD of a regular bilayer (or regular multilayer) of a 2D lattice and therefore describe the procedure we have developed for the purpose.

The treatment starts with Warren's[11] general case of randomly stacked 2D crystalline layers,

where the vector

$$\boldsymbol{R}^{k}_{m\,n\,p} = m\,\boldsymbol{a} + n\,\boldsymbol{b} + p\,\boldsymbol{c} + \boldsymbol{r}_k + \delta_p\,\boldsymbol{a} + \varepsilon_p\,\boldsymbol{b} \tag{1}$$

places an atom i with fractional coordinates $\boldsymbol{r}_i = (x_i, y_i, z_i)$ into a cell m,n in layer p and positions that layer with respect to the bottommost layer $p = 0$ by arbitrary displacements δ and ε. The electric field of the scattered wave at $\boldsymbol{Q}$ arising from the scatterer at $\boldsymbol{R}^{k}_{m\,n\,p}$ is given in electron units[5] by the usual expression

$$E = \sum_{m\,n\,p}\sum_i f_i \exp[i\,\boldsymbol{Q} \bullet \boldsymbol{R}^{i}_{m\,n\,p}]. \tag{2}$$

This is combined with (1) and converted to intensity through $I = |E|^2$. After summation over a crystalline domain of dimensions $N_a\,a$ and $N_b\,b$ it becomes

$$I = F^2 \{\sin^2 (i\,\boldsymbol{Q} \bullet N_a\,\boldsymbol{a})/\sin^2(\boldsymbol{Q} \bullet a/2)\}\{\sin^2 (i\,\boldsymbol{Q} \bullet N_b\,\boldsymbol{b})/\sin^2(i\,\boldsymbol{Q} \bullet \boldsymbol{b}/2)\}\ |\textstyle\sum_p \exp[i\,\boldsymbol{Q} \bullet (p\,\boldsymbol{c} + \delta_p\,\boldsymbol{a} + \varepsilon_p\,\boldsymbol{b})|^2 \tag{3}$$

where the structure factor F is given by

$$F^2 = |\textstyle\sum_i f_i \exp[i\,\boldsymbol{Q} \cdot \boldsymbol{r}_i]|^2 \tag{4}$$

and f_i is the atomic form factor for the i-th atom in the unit cell. The last factor in the expression (3) for I is unity for a monolayer ($p = 0$) and at the Laue scattering conditions ($i\,Q \bullet \boldsymbol{a} = h\,2\,\pi$ and $i\,\boldsymbol{Q} \bullet \boldsymbol{b} = k\,2\,\pi$). For a general 2-D lattice aligned with the laboratory x axis, the 2-D lattice vectors $\boldsymbol{a}$ and $\boldsymbol{b}$ are $\boldsymbol{a} = a\,\boldsymbol{x}$ and $\boldsymbol{b} = b \cos\theta\,\boldsymbol{x} + b \sin\theta\,\boldsymbol{y}$, with reciprocal lattice vectors $\boldsymbol{a}^*$ and $\boldsymbol{b}^*$, allowing the scattering vector $\boldsymbol{Q}$ to be related to the Miller indices (h, k) simply through $\boldsymbol{Q}_{hk} = 2\pi\,(\,h\,\boldsymbol{a}^* + k\,\boldsymbol{b}^*\,)$. At each reciprocal lattice point there is a Bragg rod extending infinitely along $\boldsymbol{Q}_z$.

For a randomly placed bilayer this last factor in the intensity expression (3) becomes

$$\sin^2 [2(Q_z\,d + 2\,\pi\,h\,\delta_1 + 2\,\pi\,k\,\varepsilon_1)]/\sin^2(Q_z\,d + 2\pi\,h\,\delta_1 + 2\,\pi\,k\,\varepsilon_1) \tag{5}$$

with the interlayer spacing d normal to the bottom layer. This factor connects the out-of-plane intensity I_{hk} with Q_z and has principal maxima at $Q_z\,d + 2\pi\,h\,\delta_1 + 2\,\pi\,k\,\varepsilon_1 = l\,2\pi$. When h and k are both zero, the out-of-plane diffraction peaks are found as usual for a crystalline material as $2\pi l/d$. However, for h and k different from zero, the out-of-plane peak positions will depend on the arbitrary displacements δ and ε and average to zero. Next, the exact position of the upper layer lattice is specified by introducing a pair of fractional lattice offsets (S_a,S_b) referenced to the unit cell edges. Diffraction peaks at $Q_z = 0$ will only be present when $2\pi\,h\,S_a + 2\pi\,k\,S_b = 2\pi\,l$. All other combinations of h and k give peaks at out-of-plane positions ($Q_z \neq 0$). The laboratory frame vector $\boldsymbol{c} = (S_a + bS_b \cos\theta\,)\,\boldsymbol{x} + bS_b \sin\theta\,\boldsymbol{y} + d\,\boldsymbol{z}$ requires that the reciprocal lattice vectors become $\boldsymbol{a}^* = (1/a)\,\boldsymbol{x} - (\cot\theta/a)\boldsymbol{y} - (S_a/d)\boldsymbol{z}$ and $\boldsymbol{b}^* = (\csc\theta/b)\boldsymbol{y} - (S_b/d)\boldsymbol{z}$. The components of $\boldsymbol{Q}_{hkl}$, which now has an additional Miller index l, referenced to the interfacial surface, are

$$\boldsymbol{Q}_{xy} = 2\pi\ [(h/a)\ \boldsymbol{x} + (k/b\ csc\ \theta + h/b\ cos\ \theta)\ \boldsymbol{y}] \tag{6}$$

$$\boldsymbol{Q}_z = 2\pi\ [l - hS_x - k\ S_y)]\ \boldsymbol{z/d} \tag{7}$$

with the magnitude

$$Q_{hkl} = (Q_{xy}^{\ 2} + Q_z^{\ 2})^{1/2} \tag{8}$$

Finally, the intensity expression used to fit the GIXD data is obtained,

$$I_{hkl} = F^2\ \{\sin^2\ (i\ \boldsymbol{Q} \bullet N_a\ \boldsymbol{a})/\sin^2(\boldsymbol{Q}_z \bullet a/2)\}\{\sin^2\ (i\ \boldsymbol{Q} \bullet N_b\ \boldsymbol{b})/\sin^2(i\ \boldsymbol{Q} \bullet \boldsymbol{b}/2)\}\{\sin^2[4\pi\ (l + hS_a + kS_b)]/\sin^2[2\pi(l + hS_a + k\ S_b)]\} \tag{9}$$

and predicts diffraction peaks at the same $\boldsymbol{Q}_{xy}$ as for a monolayer lattice but with zero or non-zero $\boldsymbol{Q}_z$ determined by the position of the second layer (S_a,S_b,d) and (hkl),

$$Q_z = 2\pi(l + hS_a + k\ S_b)/d. \tag{10}$$

There will be (h,k,l) values that make $\boldsymbol{Q}_z$ negative and correspond to a scattered wave directed below the interface and thereby absorbed, or make $\boldsymbol{Q}_z$ too large (>0.66 Å^{-1}) to fall within the detection window of the instrument. The fitting procedure identifies these peaks and excludes them from the fit. There is considerable motion at the interfacial surface at room temperature, due to capillary waves.[12] A Debye-Waller factor[5] is included in the fits to allow for an exponential decay of the intensity with increasing $\boldsymbol{Q}_{xy}$ and $\boldsymbol{Q}_z$.

Scattering into the z direction that is out of the range of the detector from bilayer electron density above the surface is then seen as one cause for the small number of observed scattering peaks in the GIXD. Another is that it is found that the best fits require that the metal ion be completely leached from the porphene layer at the interface. Removing the metal center has the effect of significantly reducing the intensity of many of the peaks. This follows for the *P4m* space group with 8-fold general positions, (x,y), (y,x), ($-x,y$), ($-y,x$), ($x,-y$), ($y,-x$), ($-x,-y$), and ($-y,-x$), which give for the structure factor,

$$F = f_M + \textstyle\sum_i f_i\ (\cos[2\pi h x_i]\cos[2\pi k y_i] + \cos[2\pi h y_i]\cos[2\pi k x_i]) \tag{11}$$

showing that if the scattering factor of the metal atom f_M vanishes, the number of zero crossings increases, thereby lowering the intensity for peaks (h,k) in their neighborhood. Finally, the observed absence of the ($0,1,l$) and ($1,0,l$) diffraction peaks in metal-free porphene results from accidental near zeroing of the above summation term for these indices due to the symmetry of atom locations in porphyrin and their approximately equal atomic numbers.

The analysis of GIXD intensities for a superlattice requires consideration of interference between the two different lattice layers by rewriting (2) as a sum of scattered fields from the two lattices, $\boldsymbol{R}^{P}$ and $\boldsymbol{R}^{SL}$,

$$I\,[\boldsymbol{Q},\boldsymbol{R}^{\mathrm{P}},\boldsymbol{R}^{\mathrm{SL}}] = |E[\boldsymbol{Q},\boldsymbol{R}^{\mathrm{P}}]+E[\boldsymbol{Q},\boldsymbol{R}^{\mathrm{SL}}]]|^2 \tag{12}$$

with

$$E[\boldsymbol{Q},\boldsymbol{R}^{\mathrm{P}}] = \textstyle\sum_{m\,n}\sum_i f_i \exp[2\,\pi\, i\, \boldsymbol{Q} \bullet \boldsymbol{R}^{i}{}_{m\,n}] \tag{13}$$

$$E[\boldsymbol{Q},\boldsymbol{R}^{\mathrm{SL}}] = \textstyle\sum_{m\,n}\sum_i f_i \exp[2\,\pi\, i\, \boldsymbol{Q}_{\mathbf{xy}} \bullet \boldsymbol{R}^{i\,SL}{}_{m\,n}]\exp(-2\pi\, i\,(hS_a + k\,S_b) + Q_z\, d\,) \tag{14}$$

which now includes their interference and interlayer separation d and gives intensity as a modulated Bragg rod with principal maxima at $Q_z = 2\pi\,[l - hS_x - S_y\,k - \mathit{\Delta}_{\mathrm{hkl}})]\,z/d$, where $\mathit{\Delta}$ is a phase correction factor given by

$$\mathit{\Delta}_{\mathrm{hkl}} = 2\pi l + \tan^{-1}\{\mathrm{Im}[E_{hkl}{}^{\mathrm{SL}*}E_{hkl}]/\mathrm{Re}[E_{hkl}{}^{\mathrm{SL}*}E_{hkl}]\} \tag{15}$$

and E_{hkl} is the value of $E[\boldsymbol{Q},\boldsymbol{R}]$ that satisfies at the Laue condition. At this point this treatment of GIXD intensity overlaps the one developed for superlattices.[9]

XR. Supplementary Figure 5GH shows specular X-ray reflectivity $R(Q_z)$ for a Zn-**2** film before (0.1 mN/m, 62 Å^2 mmA/macrocycle) and after (1.0 mN/m, 72 Å^2 mmA/macrocycle) polymerization to **1**, normalized to remove the contribution from Fresnel reflectivity $R_F(Q_z)$. $R(Q_z)$ is related to the electron density gradient d$\rho(z)$/dz,

$$R(Q_z) = R_F(Q_z)\,|\rho_\infty{}^{-1}\textstyle\int d\rho(z)/dz\,\exp(i\boldsymbol{Q}_z \bullet \boldsymbol{z})dz|^2 \tag{16}$$

where z is the distance from the top of the surface monolayer and ρ_∞ is the electron density of bulk water (0.3334 e^- / Å^3).[13] We model d$\rho(z)$/dz as a series of electron density slabs whose derivatives are non-zero only at the boundaries and are represented as a delta function times the difference of the slab electron densities.[12] Surface capillary waves arising from thermal motion at the interface effectively introduce an average roughness to any boundary parallel to the surface, treated here by smoothing the electron density gradient at the slab boundary from a delta function to a Gaussian whose FWHM is σ.

The GIXD results suggest four simple slab models for XR based on the organic bilayer primitive unit cell: the initial Langmuir film of Zn-**2** (He - tilted Zn-**2** - interlayer - tilted Zn-**2** - transition zone - subphase), superlattice models A and B (He - **1** - hydrophobic layer - transition zone - subphase) and a stacked bilayer of **1** (Model C, He - **1** - interlayer - **1** - transition zone - subphase). Fits to the observed reflectivity curves (Supplementary Figure 5GH, Supplementary Table 2) yielded the projection of electron density into the surface normal (Supplementary Table 2). The fits for Models A and C were of comparable quality and only the one for A is shown.

The XR data are in agreement with the models and fitted parameters (Supplementary Table 2) except for model B. Given the wide flexibility of the slab model, they are mostly useful for giving the total thickness of the Langmuir layer and test the compatibility of the predicted z-axis projection of the electron density of each model but with limited precision.

The architecture of the X-ray reflectivity Model A for the superlattice resembles that used for the bilayer structure of Zn-**2**. Built into the model is a critical layer of low electron density separating

the porphene lattice and the superlattice of anions.[14] The source of this low density would be a van der Waals zone between **1** and the Cl atoms in $IrCl_6^{-2}$ demonstrated in calculations of the charge density between a Stern layer and a water-mica interface.[15] The slab beneath contains the electron density of the superlattice that contains the electrons of one aquated $IrCl_6^{-2}$ anion of radius 4.04 Å and a transition zone extending to bulk water. The GIXD observations demand that 40% of the macrocycles are singly charged if the whole assembly is electroneutral. The sparse nature of such a superlattice makes it occupy only about one-fifth of the area beneath the porphene monolayer and requires a modification of equation (16) to include a second term in the Fourrier transform within the absolute value brackets: one for the transform of $d\rho(z)/dz$ traced along the z-axis from bulk water through an $IrCl_6^{-2}$ anion beneath a monolayer of **1,** described above, and the other for the transform of $d\rho(z)/dz$ from bulk water through only a monolayer of **1**, with weighting for the different occupancies. This approach accounts for the interference between the two types of reflection. This model fits the data well (Supplementary Figure 5H). The relatively electron-rich transition zone separating the anion layer and the bulk also must reflect the unevenness of the assembly, increasing the effective diameter of the superlattice anions. The total thickness of the porphene anion superlattice and the transition zone is 11.8 Å. Details are presented in Supplementary Table 2.

Model B requires that the interlayer thickness be increased by 3.5 Å. The increased overall thickness and the reduced electron density of the layers makes this model fit the data poorly for all starting conditions we tried.

Model C is similar to the bilayer model used to fit Zn-**2** except for the addition of a thick bilayer of $IrCl_6^{-2}$ anions just beneath the lower sheet of the bilayer. The expected mmA of this layer is 35 $Å^2$/macrocycle and would occupy about 50% of the surface area at the barrier position of the XR measurement. The fitting accounted for this by allowing for an interfering reflection from an empty water surface with no $IrCl_6^{-2}$ beneath it.

Supplementary Note 3

Ex Situ Polymerization Monitoring of Multilayers by IR, Raman, and UV-vis-NIR. After polymerization on water, positive charges in **1** and the $IrCl_6^{-2}$ counterions were removed by reduction with excess NaI in the subphase. Zn-**1** was then produced by adding excess $ZnCl_2$. To optimize the signal to noise ratio in our spectra, **1** and Zn-**1** were transferred to various substrates at a surface pressure high enough to transform the monolayer into small domains of bilayers and multilayers (porphite and Zn-porphite).

The course of the oxidative polymerization of Zn-**2** was followed by performing the NaI quench at various times after the reaction was launched or by varying the concentration of the oxidant at constant reaction time, and recording the IR (Main Text Figure 4) and resonant Raman (Supplementary Figure 9) intensities of peaks associated with residual CH bonds. Similar experiments were performed with Zn-**2**-d_{12}, permitting easy identification of vibrations involving CH bonds by their large isotopic shift (Supplementary Figure 10). In IR,[16,17] the characteristic CH vibrations are the stretches at 3034 cm^{-1} (C_mH) and 3110 cm^{-1}($C_\beta H$), and in Raman,[16,17] they are the in-plane bends at 1185 cm^{-1} (C_mH) and 1063 cm^{-1} ($C_\beta H$). As the size of the polymer flakes is large enough (> ~100×100 nm) for peripheral bonds to be ignored, these intensities reflect the presence of internal defects and provide information on their structure and abundance. The standard polymerization conditions (fresh 0.035 mM K_2IrCl_6 for 24 h) were chosen as those under which IR

spectra no longer showed any residual CH or CD bond above the noise level. However, Raman spectra still did, and their complete removal required 0.21 M K_2IrCl_6 and 120 hours. Spectra of the longest oxidized Zn-**1** samples are shown in Main Text Figure 3. They show little if any indication of a sharp absorbance increase at the lowest frequencies that would be associated with metallic conductivity and are qualitatively compatible with the DFT results.

IR. Main Text Figure 4 shows the stepwise conversion of Zn-**2** to **1** in a fixed interval of 3 h as a function of the concentration of the oxidant K_2IrCl_6 in the subphase. The initially present IR bands broaden as the dose is increased until they merge and become very broad absorption regions rather than distinct peaks. After very long oxidation times the bands become narrower again and reside on a flat background (Main Text Figure 4). The gradual disappearance of the CH stretching vibrations in the spectrum agrees with the proposed oxidative polymerization process and the ratio of their initial intensity in the bilayer of Zn-**2** to the level of noise under which they ultimately disappear under standard conditions suggests that in porphene prepared under these conditions there is at most one incompletely oxidized macrocycle for every 40 that have lost their protons or deuterons completely.

Main Text Figure 4 also compares the stretching and fingerprint IR regions of Zn-**1**, **1**, Zn-**2**, and **2**. The frequencies of the NH, C_mH, and $C_\beta H$ stretches in the free base of the monomer are not detectable in the Zn-**1** film. The NH stretch in **1** (3340 cm^{-1}) is shifted by about 30 cm^{-1} from its frequency in **2** (3309 cm^{-1}). The C_mH stretch is never detectable in **1**, suggesting that the polymerization occurs in the meso positions first, as would be expected.

The band at 1223 cm^{-1} in **2** has been identified as the NH deformation of the pyrrole ring.[18] The overall weak intensity in the vicinity of 1223 cm^{-1} in the IR of Zn-**2** can be used to estimate the extent of metallization of **1**. In **1** itself, containing no metal, it is high, as is that of the NH stretch at 3340 cm^{-1}, whereas in highly metallized films it is low.

The spectral contribution of free base **1** is shown as a thin line in Main Text Figure 4. The free-base macrocycles might exist in the film as localized demetallized patches or as randomly dispersed individual demetallized units, contributing to the spectral broadening. The IR spectra allow us to follow the loss and reinsertion of Zn^{2+}, using the NH stretch of **1** at 3340 cm^{-1} (cf. 3309 cm^{-1} in **2**) and NH pyrrole deformation at 1227 cm^{-1} (cf. 1223 cm^{-1} in **2**).

Resonance Raman. The Raman spectrum of Zn-**1** prepared under standard conditions is shown as the green curve in Main Text Figure 3. It contains two broad peaks at ~1350 and 1600 cm^{-1}, three smaller ones at ~ 1020, 1063 and 1454 cm^{-1}, and an indistinct shoulder at ~1190 cm^{-1}. In the spectrum of a film made from Zn-**2**-d_{12} under the same conditions (Supplementary Figure 10), the frequencies of two of the peaks are reduced by a factor of ~1.35 (Supplementary Table 4) and are assigned as sp^2-CH in-plane bending modes, in line with the previously reported[18] assignments of vibrational frequencies of Zn-**2**. The band at 1020 cm^{-1} in is assigned to the pyrrole breathing and ½ ring modes, which are not expected to be sensitive to deuterium substitution. The band at 1454 cm^{-1} also has a very small shift, expected from an secondary contribution from nearby deuterium substitution on the C_m carbon of a predominately C_mC_α mode.

The oxidative polymerization performed under standard conditions clearly is incomplete even though the IR C-H stretching bands then are so weak that they are buried in noise. Oxidative polymerization performed under more forcing conditions yields Zn-**1** films in whose Raman spectra the spectral features listed in Supplementary Table 4 gradually diminish until they, too, disappear

under noise (blue, 24 h, and orange, 72 h, both with 10 times higher oxidant concentration). The Raman spectrum of the fully healed polymer has just two strong bands at 1350 and 1600 cm^{-1} (green curve in Main Text Figure 3). Considering again the factor by which the Raman peaks of the defect-associated vibrations are reduced by oxidation that goes beyond the standard conditions and judging the level of noise under which they ultimately disappear, we conclude that there are at least 400 correctly incorporated macrocycles for each one that is defective.

The superior ability of Raman spectroscopy to detect incompletely oxidized defects can be attributed to resonance enhancement of the protiated or deuteriated defect structure. It is interesting to ask what this structure is and whether it indeed has an electronic transition close to the excitation frequency used in the Raman experiment. A clue to the structure of the incompletely oxidized defect is provided by the observation that the ratio of the relative integrated intensities observed for the C_mH and the $C_\beta H$ peaks is only about half for the defect than it is for Zn-**2**, both in the IR and the Raman spectra. This suggests that half of the four meso protons and none of the eight β protons have been lost by the defective macrocycle. The structure we propose for the defect, optimized by DFT/PBE50 for a simple model containing only nine porphyrin units, with the defective macrocycle in the center, is shown in Supplementary Figure 11. The defect carries altogether 20 CH bonds, ten on the porphyrin twisted almost perpendicular to the porphene *xy* plane and ten on its neighbors. This count and the bilayer nature of the starting Zn-**2** were taken into consideration in the evaluation of the lower limit for the ratio of properly incorporated to defective macrocycles.

UV-vis-NIR. Supplementary Figure 1 compares the absorption spectrum of Zn-**1** prepared under standard conditions and after exhaustive oxidation. There is no evidence of free-electron conductance in the spectral region examined, but the difference spectrum provides interesting information about the visible absorption associated with the defects. It has distinct peaks near ~23000 and ~15000 cm^{-1} and a vague indication of a shoulder near ~10000 cm^{-1}. This agrees with results of a TD-DFT calculation for the simple model of the defect site shown in Supplementary Figure 11. Two types of transitions characteristic of the defect would then be expected:

First, a group of four localized on the twisted macrocycle, resulting from the mixing of Soret and Q bands, two polarized roughly along *z* and two along the meso-meso axis *x* of the twisted ring (the TD-DFT/PBE50 calculated oscillator strengths of the Soret and Q bands of monomeric Zn-**2** are 3.56 and 0.016, respectively). Three of these defect-localized transitions have indeed been found in the TD-DFT output near 21000 cm^{-1} with a total oscillator strength of 1.40 (Supplementary Figure 1), and account for the strong observed absorption peak near 23000 cm^{-1}, presumably responsible for the Raman resonance (the wave number of the photons used for Raman excitation is 18 797 cm^{-1}). Their localization on the twisted ring is best seen in plots of their computed transition densities (Supplementary Figure 12) The composition of their excited state wave functions agrees with expectations for a mixture of local Soret and Q bands in the twisted macrocycle. The fourth one appears to be too weak and mixed for easy identification.

Second, transitions localized in the fused porphyrin rings surrounding the twisted macrocycle, perturbed by CH or CD termination and by interactions with the twisted porphyrin. A large number of such transitions is calculated (Supplementary Figure 1) and their computed transition densities have virtually no contribution from the twisted macrocycle, as illustrated for one of them in Supplementary Figure 12. They are mostly very weak, but a few are strong and are responsible for the absorption peaks computed at ~15000 and ~9000 cm^{-1} and observed at ~15000 nm and possibly

indistinctly also at ~10000 cm^{-1}.

Defect Density. As noted above, the level of noise in the IR spectrum and the change in the intensity of CH stretching vibrations during the standard polymerization process from clearly observable to buried in noise yield a ratio of fully fused to twisted porphyrin macrocycles of at least 40, and this corresponds to $3.5 \times 10^{12}/cm^2$. Under the most forcing conditions (0.21M K_2IrCl_6, 120 h), not even the resonant Raman spectra show any CH or CD bonds, and the defect density is at most $3.5\times10^{11}/cm^2$. If the peripheral bonds on a patch of Zn-**1** cannot be ignored, the density of these defects is even lower. However, we cannot exclude the presence of other types of defects that could be IR and Raman silent. The results for defects that we have identified compare well with defect densities observed in other 2-D materials ($10^{15}/cm^2$ in fully disordered graphene[19] and $10^{13}/cm^2$ in MoS_2[20]).

Supplementary Note 4

Electrical Conductivity. Main Text Figure 8 displays the current vs. voltage plot for Zn-**1** porphite prepared under standard conditions at various degrees of doping with I_2 from its saturated solution in hexanes. The level of doping was controled by the length of sample immersion in the solution or in pure solvent. The behavior is reversible and thorough rinsing with pure solvent returns the sample to its original non-conducting state.

The behavior found is that expected of a semiconductor, in agreement with DFT calculations with a functional containing more than ~25% of exact exchange, which yield a rectangular unit cell. There is no indication of the metallic conductivity predicted by DFT calculations with functionals containing less than 25% of exact exchange, which yield a square unit cell. Temperature-dependent measurements on a series of samples containing various concentrations of defects and various degrees of doping will be needed for full characterization of electrical conductivity of Zn-**1**.

XPS. Main Text Table 2 summarizes the observed N(1*s*) binding energies, peak widths, and peak areas determined from the fits shown in Supplementary Figure 6 with those calculated with DFT. The N(1*s*) binding energies for the cast film of free-base **2**, fitted as an overlap of two peaks at 398.2 eV (imine) and 400.2 eV (pyrrole), agree with previously reported[21] 398.0 and 400.1 eV, respectively. Their relative intensities are expected to be identical, but in reality the higher binding energy pyrrole band is about twice more intense. This has been observed before in powder samples of meso-tetra(4-carboxyphenyl)porphyrin[22] and other free-base porphyrins[23,24] prepared from acidic solutions and was attributed to adventitious partial protonation of the imine sites, with charge balance maintained by an unknown counterion.

The N(1*s*) signal in the cast film of Zn-**2** is also a pair of overlapping peaks. The position of the main peak at 398.5 eV lies in the expected range, cf. 399.3 eV for a network of m-m coupled Zn porphyrins.[25] By analogy to other metalloporphyrins,[26] the weak satellite at 400.5 eV is assigned to a shakeup associated with a $\pi\rightarrow\pi^*$ excitation. The 2.0 eV energy difference matches the excitation energy of the Q band.

In Zn-**1** the pyrrole and imine N(1*s*) levels merge into a single band at 401.0 eV, with a hint of a similar satellite peak. This striking 2 eV shift above the value of 399 eV typical of Zn porphyrins[27] places the band even above the pyrrole band of **1**, by nearly 0.3 eV. The band is shifted by 2.5 eV relative to our result for Zn-**2**, in reasonable agreement with our DFT calculated value of 2.2 eV. The shift to higher binding energies appears to be characteristic of the 2-D polymer, since

in the free base **1**, the imine and pyrrole N(1*s*) peaks are also shifted, by 1.2 and 0.5 eV, respectively. It is hardly surprising that the electrons in the more extensively conjugated **1** are capable of screening the hole better than those in **2**. The shift to higher values is also nicely compatible with the observation[28] of a broad N(1*s*) band starting at 399 eV and tailing past 402 eV for conjugated tapes obtained from nickel diphenylporphyrin by a random mix of triple (m-m + 2×β-β) and double (2×β-m) coupling. For **1**, fused regularly through exclusive m-m + 2×β-β coupling, a narrower N(1*s*) peak shifted to higher energy is reasonable. When the unit cell geometry is artificially constrained to a square and the ground state becomes metallic, the calculated shift from Zn-**2** to Zn-**1** increases to 4.7 eV, as might be expected from even easier screening. The considerable disagreement with the observed value of 2.5 eV provides an additional argument for a rectangular unit cell and against the originally predicted metallic conductivity of Zn-**1**.

Supplementary Note 5

Imaging of Multilayers. AFM. Main Text Figure 5 shows μm-sized flakes of **1** on HOPG and scores-of-μm-sized flakes of **1** on germanium, imaged by AFM. The flakes drape the HOPG steps, demonstrating their structural strength. It is clear from the conformity of the edges of some of the neighboring patches that they were torn during the transfer process.

TEM. Transmission electron microscopy of Zn-**1** reveals the detailed structure of the disordered polycrystalline material transferred from the surface to a solid substrate and shows that it is Zn-porphite, a mass of small crystalline domains of Zn-porphene multilayers. Main Text Figure 7a provides a TEM image of Zn-**1** showing overlapping lattices and moiré patterns. Main Text Figure 7b contains a 8.4 Å × 8.4 Å white square within an enlarged image of a region indicated by the white box shown in Main Text Figure 7a . The white square matches the unit cell expected for a bilayer of Zn-**1** with a (0.5,0.5) offset, different from that expected for Model C. The lattice dimensions indicate that the polymer surface orientation of this region is very nearly perpendicular to the beam. It must be only weakly attached to the rest of the porphite crystal but still with enough contact for sufficient energy dissipation. The 8.4 Å unit cell edge of Zn-**1** is similar to the edges of **1** studied by in situ GIXD.

Supplementary Figure 3A shows a flake of this material spanning a 6 μm circular hole in an Au mesh (many of the mesh holes were completely blocked by the mass of Zn-**1**). Such porphite patches are nearly opaque to the electron beam and are assumed to consist of numerous few-layer crystalline sheets of Zn-**1** whose surface normals are skewed relative to the surface normal of the supporting Au sheet. Supplementary Figure 3B contains a magnified view of a flap of Zn-**1** porphite protruding from the edge of the thicker slab at an angle. It shows a thin regular grid with lattice constants (2.96 Å, 2.67 Å, 91.4°) whose TEM contrast is determined mainly by the position of columns of Zn atoms. A comparison of these lattice constants to dimensions of the porphyrin monomer shows first, that the image is not a view of a Zn-**1** monolayer as the contrast is too strong and the sample is robust towards the 200 keV beam, hence the material must be multilayer porphite. Second, the image must be related to columns of Zn atoms that are aligned along an axis of the crystal that is not parallel to one of the faces of a simple primitive cell. Notice the second oblique lattice above the nearly square one in Supplementary Figure 3B, which must be the result of electrons traveling along a different zone axis of the porphite, its edge protruding to the side.

A piece of the interior of the flake, shown in Supplementary Figure 13G, displays many

overlapping layers of Zn-**1** and the resulting moiré patterns. Here, the microscope defocus reveals several different lattices with submolecular spacings and lattice angles in the same focal plane with high contrast. Traversing the porphite bridge under high resolution, it is possible to find thin areas or areas separated from the bulk that can be imaged and show just a bilayer (Main Text Figure 7).

Under some conditions it was possible to return to the same Zn-**1** spot after the TEM stage was rotated about its long axis. Supplementary Figure 13A-F shows the angular dependence of the same lattice at 0°, 5°, and 10° relative to an unknown orientation of the lattice at the start. The appearance of angle-dependent moiré patterns and the near invariance of the lattice constants provides visual evidence that this patch of Zn-**1** consists of only a few layers separated by a small interlayer spacing. Otherwise the lattice constants would not be invariant, since the uppermost layer would travel too far relative to the bottommost layer to maintain an unchanged column of Zn atoms over the 10° range of the rotation. A large distance from top to bottom would most likely lead to the complete disappearance of the lattice in the image as the lines of Zn atoms would no longer be parallel to the beam.

Imaging of Monolayers. As described above, at lower surface pressures, the monolayer of Zn-**1** transfers to an SiO_2 surface as islands of a monolayer, leaving some of the surface uncoated. We were unable to obtain TEM images of monolayers because the electron beam damaged the sample too rapidly, but have obtained AFM (Main Text Figure 6) and optical (Supplementary Figure 2) images of monolayer Zn-**1** on a pitted SiO_2 surface. The latter yield 3.4 Å as the sheet thickness, confirming that a monolayer is being observed. Within its more limited accuracy, AFM profilometry confirms this by providing a similar thickness of 1.1 nm for Zn-**1** as 0.4 - 1.7 nm for a monolayer of graphene.[29]

AFM. An optical microscopy (OM) image of a pitted 120 nm SiO_2 layer on Si substrate coated with a monolayer of Zn-**1** is shown in Supplementary Figure 2A. The patches of Zn-**1** appear as lighter blue against the darker gray of the uncoated substrate. Pits appear as bright white circles but some are covered with Zn-**1** and then they have a blue cast. The contrast of the film arises from reflective interference between the film and the Si substrate through the 120 nm thick transparent SiO_2 layer. This interference permitts atomically thin films to develop contrast and become observable under ordinary visible light by OM.[30,31] Main Text Figure 6 shows a collection of OM and AFM images of Zn-**1**-encased water-filled blisters and covered pits, their Raman spectra, and height profiles.

The Raman spectrum (Main Text Figure 6h) obtained through a confocal microscope confirms that the film covering the pit shown in panels 6ef is Zn-**1**. As already shown by tapping-mode (TM) AFM in Main Text Figure 5, an LB-transferred probably multilayer film of Zn-**1** will uniformly coat hundreds of μm^2 of the surface of HOPG or Ge and drape without breaking over atomic step edges with only small local perforations. The degree of expansiveness of a monolayer film can be ascertained from the TM AFM image of the region shown in Main Text Figure 6cd. The thickness of this layer ranges from about 1 nm measured by AFM profilometry (Main Text Figure 6g) at the transition from bare SiO_2 to the film of Zn-**1** (Main Text Figure 6cd). Results of measurements of the thickness of single-layer graphene (0.34 nm) by a variety of AFM methods and conditions range from 0.4 to 1.7 nm.[29] It is very likely that a monolayer of Zn-**1**, more hydrophilic through hydration of the Zn dication, will show a TM-AFM thickness range similar to or slightly larger than graphene and we conclude that the film of Zn-**1** in most regions in Main Text Figure 6cd is a monolayer or

perhaps in places at most a bilayer. Main Text Figure 6ef shows a covered pit partially filled with water. The side view of the 3D height image (Main Text Figure 6i) contrasts the observed nearly parabolic profile of the partially filled pit and the straight edges of an empty one. The covered pit partially filled with trapped water suggests that the Zn-**1** film is impermeable and stretched under tension.[32] When the tip crosses a blister as it does in Main Text Figure 6c, we believe that the blister acts as a drumhead in resonance with the oscillation of the tip. The height of the asperity may be amplified by momentary adhesion of the tip to the film of Zn-**1**. Such drumhead behavior has been observed for suspended graphene.[33] The phase of the tapping tip shows both a clear contrast between the blistered and the bare SiO_2 surfaces. By color comparison with the area above the covered pit, the phase image (Main Text Figure 6d) also shows two smaller blisters that are surrounded by thicker (2 - 4 nm) patches of multilayer Zn-**1**. The film does not always suspend itself across a pit and when this happens it is sometimes possible to observe drapes of Zn-**1** on the pit sides and/or crumpled piles at their bottoms. The final conclusion is that monolayer (or bilayer) films of Zn-**1** are covalently bonded with sufficient tensile strength for its domains to be suspended across micron-sized pits and probed without damage by forces in the TM-AFM regime.

Optical Microscopy. An OM image of the Zn-**1**-coated SiO_2/Si substrate shown in Supplementary Figure 2 is decomposed into individual red (7000 Å; panel C), green (5461 Å; panel B), and blue (4358 Å, panel A) components. The optical density is measured along a nearly identical path in the grayscale image for each color plotted in panels D-F. A cursory examination of the three grayscale images reveals that in the blue and green images regions containing the film of Zn-**1** are brighter but in the red image they are darker. The AFM images may be used as a guide for identifying the Zn-**1** covered and the bare surface regions. From these plots, the optical contrast for each of the three color wavelengths is calculated as the normalized difference between the optical density of the bare SiO_2 and a region coated by Zn-**1** at a uniform thickness. A negative contrast for the film of Zn-**1** is defined as shiny or brighter and a positive contrast is defined as darker than the bare substrate regions in the grayscale images. The contrasts calculated in this way are 0.022 at 7 000 Å, 0.013 at 5 461 Å and 0.025 at 4 358 Å. Following the analysis for single-layer graphene,[30] the wavelength dependent contrast for a monolayer of Zn-**1**, supported by 120 nm SiO_2 on Si, and under air, can be calculated similarly. Supplementary Figure 2G shows a fit to the observed contrast at the three wavelengths for a 3.4 Å thick monolayer film of **1** using a fitted real part of the film refractive index of n = 2.55 combined with the known imaginary part of the refractive index calculated from the measured absorptivity (Supplementary Figure 1) of a 20 nm thick film of **1**, whose thickness was determined by AFM. The real part of the refractive index was kept constant for all wavelengths. No adjustment was made in the contrast calculation for the numerical aperture of the microscope objective. The results are all compatible with the observation of a monolayer.

Calculated Optimized Geometries

POSCAR files for Zn porphene (Zn-1), Zn s-isoporphene, Zn z-isoporphene, and a layer of Zn-1 combined with a layer of $IrCl_6^{-2}$

Zn Porphene (Zn-**1**)

```
PBE50
   1.0000000000000000
     8.2557826517249708    0.0000000000000000   -0.0000000000000000
     0.0000000000000000   10.2395466776819397    0.0000000000000000
     0.0000000000000000    0.0000000000000000    8.3614115464422980
   C    N    Zn
    20     4     1
Direct
  0.5000000000000000  0.0000000000000000  0.9146205213456692
  0.5000000000000000 -0.0000000000000000  0.0853794786543308
  0.9143820566267961  0.0000000000000000  0.5000000000000000
  0.0856179433732040  0.0000000000000000  0.5000000000000000
  0.1683476992954637  0.0000000000000000  0.6466675704258069
  0.8316523157045340  0.0000000000000000  0.3533324295741931
  0.8316523157045340  0.0000000000000000  0.6466675704258069
  0.1683476992954637 -0.0000000000000000  0.3533324295741931
  0.0821193577394987 -0.0000000000000000  0.7971115310154445
  0.9178806642605031 -0.0000000000000000  0.2028884689845555
  0.9178806642605031  0.0000000000000000  0.7971115310154445
  0.0821193577394987  0.0000000000000000  0.2028884689845555
  0.2047162180199795  0.0000000000000000  0.9165602190648588
  0.7952838119800159 -0.0000000000000000  0.0834397809351411
  0.7952838119800159 -0.0000000000000000  0.9165602190648588
  0.2047162180199795  0.0000000000000000  0.0834397809351411
  0.3502087151163605  0.0000000000000000  0.8346783689732975
  0.6497913138836383  0.0000000000000000  0.1653216310267026
  0.6497913138836383 -0.0000000000000000  0.8346783689732975
  0.3502087151163605 -0.0000000000000000  0.1653216310267026
  0.3270092998194682 -0.0000000000000000  0.6702079253739500
  0.6729907001805316  0.0000000000000000  0.3297920746260499
  0.6729907001805316 -0.0000000000000000  0.6702079253739500
  0.3270092998194682  0.0000000000000000  0.3297920746260499
  0.5000000000000000  0.0000000000000000  0.5000000000000000
```

Zn *z*-isoporphene

```
PBE50
   1.0000000000000000
```

16.6366924618635039 -0.0000000000000000 0.0000000000000000
0.0000000000000000 8.2639952564534376 0.0000000000000000
0.0000000000000000 0.0000000000000000 10.4196790990701249
C Zn N
40 2 8
Direct
0.7500000000000000 0.0734398307498907 0.5000000000000000
0.7500000000000000 0.2472716474851353 0.5000000000000000
0.2500000000000000 0.9265601912501182 0.5000000000000000
0.2500000000000000 0.7527283675148695 0.5000000000000000
0.9587135288464808 0.6604430194541422 0.5000000000000000
0.5781120415248253 0.8142245367917553 0.5000000000000000
0.5400201413341179 0.9746230669429465 0.5000000000000000
0.6039936212760182 0.0816503686084147 0.5000000000000000
0.6753057063879524 0.9938532172818881 0.5000000000000000
0.9162261135917831 0.5135111815473152 0.5000000000000000
0.9568753885754546 0.3625800609415037 0.5000000000000000
0.8961289532407619 0.2483423102993711 0.5000000000000000
0.8230302797416940 0.3314080543960145 0.5000000000000000
0.5412864711535192 0.6604430194541422 0.5000000000000000
0.9218879584751747 0.8142245367917553 0.5000000000000000
0.9599798586658821 0.9746230669429465 0.5000000000000000
0.8960063787239818 0.0816503686084147 0.5000000000000000
0.8246942936120476 0.9938532172818881 0.5000000000000000
0.5837738864082169 0.5135111815473152 0.5000000000000000
0.5431246114245454 0.3625800609415037 0.5000000000000000
0.6038710467592381 0.2483423102993711 0.5000000000000000
0.6769697202583060 0.3314080543960145 0.5000000000000000
0.0412864641535222 0.3395570095458638 0.5000000000000000
0.4218879584751747 0.1857754332082422 0.5000000000000000
0.4599798286658796 0.0253769480570583 0.5000000000000000
0.3960063787239818 0.9183496543915837 0.5000000000000000
0.3246942936120476 0.0061468077181246 0.5000000000000000
0.0837739014082146 0.4864888184526777 0.5000000000000000
0.0431245854245503 0.6374199390584963 0.5000000000000000
0.1038710167592427 0.7516576897006289 0.5000000000000000
0.1769697352583037 0.6685919456039855 0.5000000000000000
0.4587135288464808 0.3395570095458638 0.5000000000000000
0.0781120345248212 0.1857754332082422 0.5000000000000000
0.0400201603341159 0.0253769480570583 0.5000000000000000
0.1039936062760205 0.9183496543915837 0.5000000000000000
0.1753057063879524 0.0061468077181246 0.5000000000000000
0.4162260835917877 0.4864888184526777 0.5000000000000000

0.4568754175754535 0.6374199390584963 0.5000000000000000
0.3961289832407573 0.7516576897006289 0.5000000000000000
0.3230302797416940 0.6685919456039855 0.5000000000000000
0.7500000000000000 0.6640646573988391 0.5000000000000000
0.2500000000000000 0.3359353426011609 0.5000000000000000
0.6590504124930519 0.8331036000170968 0.5000000000000000
0.8354748702944619 0.4924598129097006 0.5000000000000000
0.8409495875069481 0.8331036000170968 0.5000000000000000
0.6645251297055381 0.4924598129097006 0.5000000000000000
0.3409496175069506 0.1668963699829007 0.5000000000000000
0.1645251437055322 0.5075401870902922 0.5000000000000000
0.1590503974930471 0.1668963699829007 0.5000000000000000
0.3354748412944630 0.5075401870902922 0.5000000000000000

Zn *s*-isoporphene
PBE50
1.0000000000000000
8.7190488891854496 0.0000000000000000 -0.0469250786346632
0.0000000000000000 10.4031473860487154 0.0000000000000000
2.5152336377453297 0.0000000000000000 7.8753802543446101
C N Zn
20 4 1
Direct
0.9984270745741802 0.5000000000000000 0.4133855572582661
0.0015729254258197 0.5000000000000000 0.5866144427417338
0.4179666001257655 0.5000000000000000 0.8663618386926757
0.5820333998742273 0.5000000000000000 0.1336381463073268
0.6552070919347264 0.5000000000000000 0.2651926259144701
0.3447929080652737 0.5000000000000000 0.7348074040855324
0.5797173091755123 0.5000000000000000 0.4497555199116531
0.4202826908244875 0.5000000000000000 0.5502444800883469
0.7074617594289759 0.5000000000000000 0.5160140636981836
0.2925382405710240 0.5000000000000000 0.4839859663018261
0.8490596997801640 0.5000000000000000 0.3826079538437131
0.1509403002198360 0.5000000000000000 0.6173920751562927
0.3317158813168128 0.5000000000000000 0.0393647672512948
0.6682841186831872 0.5000000000000000 0.9606352447487062
0.4129406614343244 0.5000000000000000 0.1645696257083819
0.5870593385656757 0.5000000000000000 0.8354303892916229
0.2914567134339575 0.5000000000000000 0.3175247717201302
0.7085432865660424 0.5000000000000000 0.6824752282798698
0.1447438808941603 0.5000000000000000 0.2817004886799396
0.8552561191058398 0.5000000000000000 0.7182995113200604

0.8157741812064233 0.5000000000000000 0.2322060226863929
0.1842258187935767 0.5000000000000000 0.7677940073136096
0.1693962647017588 0.5000000000000000 0.1129447926300774
0.8306037352982414 0.5000000000000000 0.8870551773699201
-0.0000000000000000 0.5000000000000000 -0.0000000000000000

Electroneutral combination of a layer of Zn-**1** with a layer of $IrCl_6^{-2}$ (Figure 2)
PBE
1.0000000000000000
18.8032906982880910 0.0720388031660995 -0.0018451120342904
0.0770617014095911 20.0438934832805025 -0.0359375725188315
-0.0222090326223448 -0.0337468039118333 18.8357239005970030
C Cl H N Ir
100 6 10 20 1
Direct
0.0023491065155914 0.6200680985489144 0.7076606479318516
0.9977064823739500 0.6193613017142798 0.2927721715502823
0.0262765449358518 0.6170404889595653 0.6356563751213630
0.9738090955452715 0.6165340802612389 0.3647499028026259
0.0068579374767397 0.6265709459061196 0.1627107139572412
0.9930136989051400 0.6270720282301553 0.8378844831797525
0.8360034509779526 0.6272015937845545 0.0083864281921928
0.1639573827833796 0.6275149832611331 0.9920562681650432
0.8928169591514136 0.6223137724553557 0.2025481653742207
0.1071375711911953 0.6232079430217098 0.7978864460858697
0.8969813227121359 0.6265038432830867 0.1260116260842473
0.1028854176440794 0.6271283846009114 0.8744557097692212
0.9643414695149780 0.6223713289103437 0.2264134727166910
0.0356805787910503 0.6231232240663601 0.7740245440943010
0.8346590601746174 0.6257271726982582 0.0831873494277028
0.1652599224622813 0.6266412937708717 0.9172220185610181
0.4360253137467459 0.6155187511987776 0.2083518971648516
0.5640846342081463 0.6157707058370915 0.7918879735345452
0.3978790686123073 0.6174525394293015 0.0927740247185937
0.6021998088736880 0.6173950954254537 0.9074673235000470
0.7640883771000063 0.6194521719733951 0.1917825110010684
0.2359457022010308 0.6205368582212927 0.8085259089169406
0.8023101857729790 0.6167005513149091 0.3075072629328827
0.1977575373947828 0.6173156672381258 0.6928563094774364
0.6069261435775228 0.6137969229287942 0.3622912005178414
0.3931917050262412 0.6140240096971927 0.6379761345182056
0.4929671954205042 0.6135946977786394 0.4024250683188252
0.5071464727839494 0.6137822281300993 0.5978129119396380

0.5932326950404098	0.6167779762136261	0.0377075203871832
0.4068466655619771	0.6172838282595102	0.9624986200944903
0.4970941879030618	0.6139994222051420	0.3257955107059132
0.5030268586452744	0.6143012194869223	0.6744493681672955
0.5642824498770219	0.6134757770463315	0.4261517665035311
0.4358347756024146	0.6135605514745868	0.5740939175773443
0.7029946654653918	0.6202258873473696	0.0744445269792319
0.2970241319027589	0.6212663503331861	0.9258654563983385
0.4738367848752842	0.6157812557063486	0.0951742293961429
0.5262617137343860	0.6159024745256519	0.9050523667347241
0.3738646265072036	0.6174027154866394	0.1646854250716235
0.6262216468753463	0.6174911792633240	0.8355651983268189
0.7264225879993870	0.6155217795150670	0.3049884837196082
0.2737074245154304	0.6160849386800333	0.6953375333040915
0.8263542363391950	0.6193927283096164	0.2355759323454453
0.1736901026373810	0.6203002463088899	0.7647794875333130
0.5176517502867843	0.6157937110226130	0.0344712295247433
0.4824530871156828	0.6159571010405012	0.9657503616977825
0.6825261133563397	0.6142114106049645	0.3656042536674491
0.3175859980213318	0.6144620504090172	0.6346966700932956
0.4346156337920684	0.6147528006879865	0.2831627236261269
0.5654974505830235	0.6150654272092232	0.7170774869114239
0.7654969953835795	0.6218355600690192	0.1170461660923954
0.2345123997702274	0.6230164916434127	0.8832874533628650
0.6361752674497436	0.6144964331735815	0.6082884547663567
0.3639262483047073	0.6141779122616238	0.3919634556698294
0.5978391141639218	0.6135492579469385	0.4927522529646992
0.4022729399125471	0.6134752401212424	0.5075029389056024
0.9640956832793414	0.6156950912041826	0.5920804474210621
0.0360045059503537	0.6152892644136416	0.4083145714265202
0.8069863556270007	0.6213539423896358	0.7624873161325457
0.1930143167501951	0.6205864195855130	0.2378471161555316
0.6929481089067301	0.6188829000998799	0.8024976474435009
0.3071259731828830	0.6186160829025870	0.1977998536045220
0.7931309727646201	0.6145485312147929	0.4377925927195750
0.2069746042448858	0.6145628321866554	0.5625391490670445
0.9071365005754979	0.6156150143801831	0.3978713143536159
0.0929505290929492	0.6159238413313618	0.6025038575921910
0.6972104092523133	0.6173774199905956	0.7259134736817112
0.3028972660441722	0.6169604544048509	0.2743274707700551
0.7642437303232985	0.6218661488643902	0.8263711328896934
0.2357314701690567	0.6213510369226354	0.1739473094329826
0.9029353707706568	0.6145211215571142	0.4745126984758954

0.0971511166322650	0.6145083181411171	0.5258561383259036
0.8357795522010021	0.6156939655700386	0.3740292019208418
0.1643171771645704	0.6160190207545859	0.6263211222807576
0.6737850052101964	0.6138652303009371	0.4950703159005264
0.3263114185977579	0.6137106331779368	0.5051939245266117
0.5739427093548047	0.6138254286386131	0.5646478014013426
0.4261638033496881	0.6136385979624176	0.4355988082881447
0.9262739347478621	0.6202070599004711	0.7052934552859534
0.0737642067931426	0.6194649350911611	0.2951223676875034
0.7175748940021822	0.6140780799322585	0.4343788690640184
0.2825350233452746	0.6140811217445626	0.5659020408810163
0.8825063972181065	0.6229448271172457	0.7659422641845175
0.1174396835251574	0.6220324454487864	0.2344600475071762
0.6348499288773874	0.6154576572731958	0.6831551599475825
0.3652624108975607	0.6151146105973522	0.3170949659646982
0.9654048485443540	0.6144606420398723	0.5172155422422923
0.0346819866067689	0.6143364892092040	0.4831730583251232
0.7976806898958193	0.6247818800222584	0.8927483171251539
0.2022516859188335	0.6243268678234383	0.1076129397301369
0.8736062746878795	0.6279236092669100	0.8952485232408951
0.1261725755907856	0.6269643416682918	0.1051464494321031
0.7736819734070766	0.6242916396143919	0.9647300837384122
0.2262544269342604	0.6246633604279224	0.0356340066170535
0.9174635049683969	0.6266932389335710	0.8346178435938287
0.0823794691821592	0.6257523707687733	0.1658376900397218
0.7071564755975113	0.6210375095727529	0.9977040289887641
0.2928816385995172	0.6217616156136676	0.0025991414280891
0.6357249893140960	0.6183698763080826	0.9739848409661269
0.3643062147365888	0.6187786646083071	0.0262796744511937
0.0929020979032372	0.4799081897558373	0.9658709679753256
0.9214867611449850	0.4751248874423239	0.9303439514061569
0.0237137757665286	0.3474711514549376	0.8944058769813250
0.9766409707039501	0.4733743135324769	0.0956816063791628
0.0786392447586965	0.3442852523287626	0.0601538083531823
0.9078509295880836	0.3391161550818414	0.0240877742273943
0.0540815531508429	0.6271537019620727	0.0181648644082849
0.9458227234128690	0.6279466705133905	0.9820367656924586
0.6540933134049173	0.6163702533147405	0.2177456212418666
0.3460141059617145	0.6172194811520789	0.7825588682885598
0.5461667653594328	0.6137406729541477	0.1823738037421811
0.4539661215321553	0.6141550579868442	0.8178431333703685
0.8538911547452563	0.6166296185025917	0.6185031356889428
0.1461938846530432	0.6161048277190354	0.3819465529653757

0.7463570979071695	0.6148183635816443	0.5819209252779854
0.2537491150594736	0.6144469005701247	0.4183253971214959
0.9657391149757605	0.6300380632325251	0.1027321554984669
0.0339542356630747	0.6301615783913223	0.8978027590775111
0.4947130149804148	0.6147646995148411	0.1650426226981893
0.5054063764212219	0.6150422203941605	0.8351780793221645
0.7055824456620647	0.6168861913953443	0.2350175449636620
0.2945312394097920	0.6177693201887378	0.7652719918702128
0.5659732157541021	0.6140893598980988	0.3020762939366122
0.4341511446889176	0.6144366514623824	0.6981746572941139
0.6343272392114423	0.6176033250088087	0.0980118836528524
0.3657749546407326	0.6184892036887303	0.9022424609533717
0.6947995325304891	0.6144176862934294	0.5649115209864126
0.3053011834696732	0.6141145748088305	0.4353484467060156
0.9054400181893172	0.6172925457759196	0.6355628550217785
0.0946459829844955	0.6167275430865621	0.3648549284143814
0.7662057551039029	0.6186393314833853	0.7023577876455434
0.2338464316497257	0.6179545495911526	0.2979099097978752
0.8340450029882153	0.6141448553859188	0.4980578945654238
0.1660359108574118	0.6139445404442607	0.5022839571932975
0.8942715739350835	0.6303801508630730	0.9650589109214690
0.1054864125051087	0.6296206712564369	0.0354901581009175
0.0002428789963896	0.4088908730086852	0.9954119749256060

Supplementary References

1. Bull, R. A. & Bulkowski, J. E. Tetraphenylporphyrin monolayers: Formation at the air-water interface and characterization on glass supports by absorption and fluorescence spectroscopy. *J. Colloid and Interface Sci.* **93**, 1-12 (1983).

2. Roberts, G. G. Ed. *Langmuir-Blodgett Films*. (Plenum Press: 1990).

3. Mansur, H., de Sales, N. F., & Mansur, A. A. P. Preparation and characterization of 5,10,15,20-tetraphenylporphyrin Langmuir films for gas sensor applications" *Surf. Interfacce Anal.* **43**, 1423-1429 (2011).

4. Jentzen, W., Shelnutt, J. A., & Scheidt, W. R. Metalloporphenes: dimers and trimers. *Inorg. Chem.* **55**, 6294-6299 (2016).

5. Warren, B. E. *X-ray Diffraction*. (Dover Publications, Inc.: 1969).

6. Lesieur, P., Vanevyver, M., Ruaudel-Teixier, B., & Barraud, A. Orientational studies of langmuir-blodgett films of porphyrins with polarized resonant Raman spectroscopy. *Thin Solid Films* **159**, 315-322 (1988).

7. Tan, J., Li, W., He, X., & Zhao, M. Stable ferromagnetism and half-metallicity in two-dimensional polyporphyrin frameworks. *RSC Adv.* **3**, 7016-7022 (2013).

8. Uysal, A., Rock, W., Quiao, B., Bu, W., & Lin, B. Two-step adsorption of $PtCl_6^{2-}$ at a charged Langmuir monolayer: Role of hydration and ion correlations. *J. Phys. Chem. C* **121**, 25377-25383 (2017).

9. Kmetko, J., Datta, A., Evmenenko, G., & Dutta, P. The effects of divalent ions on Langmuir monolayer and subphase structure: A grazing-incidence diffraction and Bragg Rod study. *J. Phys. Chem. B* **105**, 10818-10825 (2001).

10. Jain, A., Ong, S. P., Hautier, G., Chen, W., Richards, W. D., Dacek, S., Cholia, S., Gunter, D., Skinner, D., Ceder, G., & Persson, K. A. The materials project: A materials genome approach to accelerating materials innovation. *APL Mater.* **1**, 011002 (2013).

11. Warren, B. E. X-ray diffraction in random layer lattices. *Phys. Rev.* **59**, 693-698 (1941).

12. Bu,W. & Schlossman, M. L. "Synchrotron X-ray scattering from liquid surfaces and interfaces." In *Synchrotron Light Sources and Free-Electron Lasers*, Jaeschke, E., Khan, S., Schneider, J, & Hastings, J., Eds. (Springer, 2016), pp. 1579-1616.

13. Tomberli, B., Benmore, C. J., Egelstaff, P. A., Neuefeind, J., & Honkimäki, V. Isotopic quantum effects in water structure measured with high energy photon diffraction. *J. Phys.: Condens.. Matter* **12**, 2597-2612 (2000).

14. Mezger, M., Sedlmeier, F., Horinek, D., Reichert, H., Pontoni, D., & Dosch, H. On the origin of the hydrophobic water gap: An X-ray reflectivity and MD simulation study. *J. Am. Chem. Soc.* **132**, 6735-6741 (2010).

15. Bourg, I. C., Lee, S. S., Fenter, P., & Tournassat, C. Stern layer structure and eregetics at mica-water interfaces. *J. Phys. Chem. C* **121**, 9402-9412 (2017).

16. Li, X. Y. & Zgierski, M. Z. Porphine force field: in-plane normal modes of free-base porphine; comparison with metalloporphines and structural implications. *J. Phys. Chem.* **95**, 4268-4287 (1991).

17. Jarzęcki, A. A., Kozlowski, P. M., Pulay, P., Ye, B. H., Li, X. Y. Scaled quantum mechanical and experimental vibrational spectra of magnesium and zinc porphyrins. *Spectrochim. Acta A Mol. Biomol. Spectrosc.* **53**, 1195-1209 (1997).

18. Li, X. Y. & Zgierski, M. Z. Porphine force field: in-plane normal modes of free-base porphine: Comparison with metalloporphines and structural implications. *J. Phys. Chem.* **95**, 4268-4287 (1991).

19. Jorio, A., et al. Raman study of ion-induced defects in *N*-layer graphene *J. Phys. Condens. Matter.* **22**, 334204 (2010).

20. Yang, L., et al. Chloride molecular doping technique on 2D materials: WS_2 and MoS_2 *Nano Lett.* **14**, 6275-6280 (2014).

21. Ghosh, A., Almlöf, J., & Gassman, P. G. *Ab initio* SCF studied of basis set effects in free base porphin. *Chem. Phys. Lett.* **186**, 113-118 (1991).

22. Yamashige, H., Matsuo, S., Kurisaki, T., Perera, R. C. C., & Wakita, H. Local structure of nitrogen atoms in a porphine ring of *meso*-phenyl substituted porphyrin with an electron-withdrawing group using X-ray photoelectron spectroscopy and X-ray absorption spectroscopy. *Anal. Sci.* **21**, 635-639 (2005).

23. Friesen, B. A., Wiggins, B., McHale, J. L., Mazur, U., & Hipps, K. W. A self-assembled two-dimensional zwitterionic structure: H_6TSPP studied on graphite. *J. Phys . Chem. C* **115**, 3990-3999 (2011).

24. Eskelsen, J. R., Wang, Y., Qui, Y., Ray, M., Handlin, M., Hipps, K. W., & Mazur, U. Protonation state of core nitrogrens in the meso-tetra(4-carboxyphenyl)porphrin impacts the chemical and physical properties of nanostructures formed in acid solutions. *J. Porphyrins and Phthalocyanines* **16**, 1233-1243 (2012).

25. Chen, A., Zhang, Y., Chen, J., Chen, L., & Yu, Y. Metallopropyrin-based organic polymers for carbon dioxide fixation to cyclic carbonate. *J. Mat. Chem. A* **3**, 9807-9816 (2015).

26. Niwa, Y., Kobayashi, H., & Tsuchiya, T. X-ray photoelectron spectroscopy of tetraphenylporphin and phthalocyanine. *J. Chem. Phys.* **60**, 799-807 (1974).

27. Polzonetti, G., Ferri, A., Russo, M. V., Iucci, G., Licoccia, S., & Paolesse, R. Platinum complex/Zn-porphyrin macrosystem assemblies: Electronic structure and conformational investigation by x-ray photoelectron spectroscopy. *J. Vac. Sci. Technol. A* **17**, 832-839 (1999).

28. Bengasi, G., Baba, K., Frache, G., Desport, J., Gratia, P., Heinze, K., & Boscher, N. Conductive fused porphyrin tapes on sensitive substrates by a chemical vapor deposition approach. *Angew. Chem.* **58**, 2125-2130 (2019).

29. Shearer, C. J., Slattery, A. D., Stapleton, A. J., Shapter, J. G., Gibson, C. T. Accurate thickness measurement of graphene. *Nanotechnology* **27**, 125704-125714 (2016).

30. Blake, P., Hill, E. W., Castro Neto, A. H., Novoselov, K. S., Jiang, D., Yang, R., Booth, T. J., Geim, A. K. Making graphene visible. *Appl. Phys. Lett.* **91**, 63124-63127 (2007).

31. Casiraghi, C., Harschuh, A., Lidorikis, R., Quian, H., Harutyunyan, H., Gokus, T., Novoselov, K. S., Ferrari, A. C. Rayleigh Imaging of Graphene and Garphene Layers. *Nano Lett.* **7**, 2711-2717 (2007).

32. Bunch, J. S., Verbridge, S. S., Alden, J. S., van der Znade, A. M., Parpia, J. M., Craighead, H. G., McEuen, P. L. Impermeable Atomic Membranes from Graphene Sheets. *Nano Lett.* **8**, 2458-2462 (2008).

33. Klimov, N. N., Jung, S., Zhu, S., Li, T., Wright, C. A., Solares, S. D., Newell, D. B., Zhitenev, N. B., Stroscio, J. A. Electromechanical properties of graphene drumheads. *Sci.* **336**, 1557-1561 (2012).
